# Regulation of the Zinc Deficiency Response in the Legume Model *Medicago truncatula*

**DOI:** 10.3389/fpls.2022.916168

**Published:** 2022-06-30

**Authors:** Feixue Liao, Grmay Hailu Lilay, Pedro Humberto Castro, Herlander Azevedo, Ana G. L. Assunção

**Affiliations:** ^1^Department of Plant and Environmental Sciences, University of Copenhagen, Frederiksberg, Denmark; ^2^CIBIO-InBIO Research Centre in Biodiversity and Genetic Resources, University of Porto, Vairão, Portugal; ^3^BIOPOLIS Biodiversity and Land Planning, Vairão, Portugal; ^4^Departamento de Biologia, Faculdade de Ciências, Universidade do Porto, Porto, Portugal

**Keywords:** *Medicago truncatula* (Medicago), Zn deficiency response, F-bZIP transcription factors, ZIP transporters, nicotianamine (NA), legume

## Abstract

The zinc deficiency response in *Arabidopsis thaliana* is regulated by F-group basic region leucine-zipper (F-bZIP) transcription factors, and there is evidence of evolutionary conservation of this regulatory network in land plants. Fundamental knowledge on the zinc homeostasis regulation in crop species will contribute to improving their zinc nutritional value. Legumes are protein-rich crops, used worldwide as part of traditional diets and as animal forage, being therefore a good target for micronutrient biofortification. Here, we identified F-bZIP transcription factors in representative legume species and functionally characterized the two F-bZIPs from *Medicago truncatula*. Results indicate that MtFbZIP1 is the functional homolog of *A. thaliana* bZIP19 and bZIP23, while MtFbZIP2 does not play a role in the zinc deficiency response. Additionally, analysis of *M. truncatula* genes from the Zrt/Irt-like protein (ZIP) family of zinc transporters or encoding nicotianamine synthase enzymes that produce the zinc ligand nicotianamine, support the conservation of the F-bZIP-regulated zinc deficiency response in *M. truncatula*. Phylogenetic analysis of F-bZIP homologs enriched in legume species reinforces the branching into two groups, with MtFbZIP1 and MtFbZIP2 mapping in Groups 1 and 2, respectively. This phylogeny combined with the functional characterization of MtFbZIPs supports the suggested conservation of the zinc deficiency response associated with Group 1 F-bZIPs, and the more variable evolutionary paths associated with Group 2. Overall, we provide novel insight on the mechanisms of response to zinc deficiency in *M. truncatula*, which contributes to developing strategies for improving zinc content in legume crops.

## Introduction

In biological systems, the importance of zinc (Zn) as an essential micronutrient relates with its requirement as a key structural and catalytic component for a large number of proteins (Maret and Li, 2009). It is estimated that Zn-binding proteins represent nearly 10% of the proteome in eukaryotes, and it is the second most abundant transition metal in living organisms, after iron (Fe) (Andreini et al., 2006). A large number of proteins have structural Zn sites, such as the Zn-finger transcription factors, but catalytic Zn sites are also frequent, being present in all six enzyme classes including key metabolic enzymes such as carbonic anhydrase, alkaline phosphatase and Cu/Zn superoxide dismutase (Vallee and Auld, 1990; Colvin et al., 2010). Micronutrient Zn acquisition, distribution and intracellular availability rely on membrane transporters and low-molecular-weight ligands, which are part of the Zn homeostasis network. This network is tightly regulated to avoid Zn deficiency or toxicity (Clemens, 2001; Sinclair and Krämer, 2012). In *Arabidopsis thaliana* (Arabidopsis), the Zn homeostasis regulators bZIP19 and bZIP23 are basic region leucine-zipper transcription factors from the F group (F-bZIP), which play a central role in the transcriptional regulation of the Zn deficiency response. They bind to Zinc Deficiency Response Elements (ZDRE) in the promoters of target genes, which are activated under Zn deficiency (Assunção et al., 2010). The target genes comprise a small set of genes involved in Zn transport and distribution. These include genes encoding Zn transporters from the Zrt/Irt-like protein (ZIP) family, which mediate Zn uptake into the cell, and nicotianamine synthase (NAS) enzymes that produce the low-molecular-weight Zn ligand nicotianamine (NA), which is involved in Zn intercellular and long-distance movement (Guerinot, 2000; Clemens et al., 2013). The *bzip19 bzip23* double mutant (*bzip19/23*) is hypersensitive to Zn deficiency and shows no visible phenotype at Zn sufficiency (Assunção et al., 2010). The bZIP19 and bZIP23 transcription factors not only function as key regulators of the Zn deficiency response, but also as direct sensors of the intracellular Zn concentration, through direct binding of Zn^2+^ ions to the transcription factor’s Zn-Sensor Motif (ZSM) (Lilay et al., 2021).

There is evidence for the evolutionary conservation of F-bZIP transcription factors and the Zn deficiency response across land plants (Castro et al., 2017). This is supported by functional analysis of F-bZIP homologs from cereals, i.e., barley, wheat and rice (Evens et al., 2017; Nazri et al., 2017; Lilay et al., 2020). These studies support a conserved mechanism of Zn deficiency response anchored at functional homologs of the Arabidopsis bZIP19 and bZIP23 transcription factors. Furthermore, there is evidence from Arabidopsis that the activity of F-bZIP transcription factors can be modulated through modifications in their ZSM to increase plant and seed Zn content (Lilay et al., 2021). Therefore, exploring such conservation can help unravel the Zn homeostasis network and its regulation in other plant species, and contribute to the development of strategies that target increased Zn nutritional value in crops (biofortification).

Zn deficiency in agricultural soils is widespread and about one-third of the world’s human population is at high risk of Zn malnutrition, mainly populations depending on cereal-rich diets (Wessells and Brown, 2012). Legume crops are characterized by their high nutritional value as an important source of protein, but also fiber, oil, phytochemicals and micronutrients Fe and Zn (Beebe et al., 2000; Castro-Guerrero et al., 2016; Robinson et al., 2019). Legume pulse crops are present in traditional diets throughout the world, and are considered an affordable and important component of healthy diets. Also legume forages are used worldwide for animal feed (Castro-Guerrero et al., 2016). The high protein content of legume plants is related with their ability to establish symbiotic associations with nitrogen-fixing bacteria (rhizobia), which is also the basis for legume use in crop rotation strategies, as an alternative to synthetic nitrogen fertilizers (Escudero et al., 2020). Moreover, these are broadly resilient crops, able to colonize marginal land and nutrient-poor soils (Ibrahim and Ramadan, 2015). Therefore, legume species are considered important targets for micronutrient Fe and Zn biofortification efforts, to help tackle these micronutrient deficiencies in human diets (Kumar and Pandey, 2020).^1^

In recent years, the mechanisms of Zn homeostasis in the model legume *Medicago truncatula* are being unraveled, with the identification of members from different metal micronutrient transporter families, including the ZIP transporters, and the metal ligand producing enzymes NAS. Studies have particularly targeted the transport and delivery of Zn and other metal micronutrients to the nodule-rhizobia infected cells (López-Millán et al., 2004; Avenhaus et al., 2016; Abreu et al., 2017; Escudero et al., 2020). In legume species, this is an important component of the plant metal micronutrient homeostasis, with a significant allocation to the symbiosome, where an adequate metal delivery and availability is essential for metalloprotein requirements and a proper symbiotic nitrogen fixation (Abreu et al., 2020). Here, considering the evolutionary conservation of the F-bZIP transcription factors (Castro et al., 2017), we investigated the Zn deficiency response in the legume model *M. truncatula.* The F-bZIP homologs from *M. truncatula* and other legume species were identified and the *M. truncatula* F-bZIP regulatory network was investigated. This knowledge contributes to a deeper understanding of the mechanisms of Zn homeostasis regulation in *M. truncatula* and legume species in general.

## Materials and Methods

### Phylogenetic Analysis

Phylogeny of F-bZIP genes was determined as previously reported (Castro et al., 2017). The amino acid sequences of F-bZIPs were retrieved from multiple databases (Plaza Dicots 4.5^2^; Phytozome v13^3^; Legume Information System^4^), and included the following species: *Arachis ipaensis, Glycine max, Lotus japonicus*, *Lupinus angustifolius, Phaseolus vulgaris, Pisum sativum*, and *Trifolium pratense*. Due to incomplete F-bZIP assignment in the above mentioned databases, F-bZIPs from *Cajanus cajan, Cicer arietinum, Medicago truncatula* and *Vigna radiata var. radiate* were derived *via* NCBI blastp,^5^ with default settings. For an evolutionary contextualization, the analysis also included other species as representatives of major plant taxa, namely a bryophyte (*Physcomitrella patens*), a pteridophyte (*Selaginella moellendorffii*), the basal angiosperm (*Amborella trichopoda*), a model monocot (*Oryza sativa*), the model angiosperm eudicot (*A. thaliana*), and four species from the clade Fabids, which are not from the Fabaceae family (*Prunus persica*, *Cucumis melo*, *Cucumis sativus*, and *Citrullus lanatus*). Sequence information is summarized in Supplementary Table 1. The phylogenetic analysis was performed on CIPRES Science Gateway V3.3^6^ (Miller et al., 2011). The sequence alignment was performed with MAFFT v.7471 and tree computation was performed with RAxML-HPC v.8, inputting 1000 bootstrap iterations, as described by Castro et al. (2017). The phylogenetic tree was visualized *via* SeaView Version 4 software (Gouy et al., 2010). For the gene enrichment analysis, the phylogenetic relationship between species was established based on the information available in the Dicots Plaza database and published by Champagne et al. (2007).

The ZIP phylogenetic analysis was performed with *M. truncatula*, *A. thaliana*, and *A. trichopoda ZIP* family members to infer on their relationship. The sequences used in the analysis were retrieved from the Plaza Dicots 4.5 database and TAIR, The Arabidopsis Information Resource.^7^ The *M. truncatula* genes Medtr3g081630 and Medtr4g006710 were annotated as *MtZIP17* and *MtZIP18*, respectivly. The sequences used in the analysis are summarized in Supplementary Table 2. The amino acid sequences were aligned and an unrooted phylogenetic analysis was performed with the neighbor-joining method (Jukes-Cantor model with 1,000 bootstrap replicates), using the CLC Main Workbench 8.0.1 software.

A motif search was performed on the gene promoter sequences of the *M. truncatula*, *A. thaliana*, and *A. trichopoda ZIP* genes previously incorporated in the unrooted tree. This analysis was extended to the four annotated *M. truncatula NAS* genes (Escudero et al., 2020), which were retrieved from the Plaza Dicots 4.5 database (Supplementary Table 2). Promoter regions were downloaded in FASTA format from the Sequence retrieval feature of the Plaza database, and consisted of the –2 kb span, or the intergenic region when in the presence of a gene within the –2 kb span. To search for the presence of 10-bp ZDRE motifs, the consensus sequence (RTGWCGACAY; A/GTGT/ACGACAC/T) (Castro et al., 2017) was used and the MEME Suite 5.3.3^8^ and Motif Alignment & Search Tool (MAST v4.11.2) software were employed. Only motifs with a position p-value inferior to 0.0001 were selected. One mismatch from the consensus sequence was allowed.

### Plasmid Construction and Plant Transformation

The *pCaMV35S:MtFbZIP1-CFP-HA* and *pCaMV35S:MtFbZIP2-CFP-HA* constructs for stable transformation of the Arabidopsis *bzip19/23* double mutant and *bzip19/23-pZIP4:GUS* reporter line, respectively, were generated as follows: the full-length coding sequence (CDS) of *MtFbZIP1* (MtrunA17_Chr4g0036971) and *MtFbZIP2* (MtrunA17_Chr3g0090531) were amplified from the cDNA of *M. truncatula* using, for MtFbZIP1, forward and reverse primers containing *Not*I and *Asc*I restriction sites, respectively, and for MtFbZIP2, forward and reverse primers designed for TOPO^®^ reaction (Supplementary Table 3). The *MtFbZIP1* and *MtFbZIP2* were then cloned into pENTR™-D-TOPO^®^ vector by restriction-ligation and TOPO reaction, respectively. The cloning into pEarleyGate-102 Gateway vector (Earley et al., 2006), carrying a *Cauliflower mosaic virus* (CaMV) 35S promoter, a C-terminal cyan fluorescent protein (CFP), and a HA-tag, the transformation into *Agrobacterium tumefaciens*, and generation of Arabidopsis mutant lines were performed as described by Lilay et al. (2019). Transgenic plants were selected for Basta (phosphinothricin) resistance. For *MtFbZIP1*, homozygous transgenic seeds (T3 generation) of three independently transformed lines were selected. The expression of *MtFbZIP1* in each line was confirmed by real-time quantitative reverse transcription–PCR (RT–qPCR). The lines were referred to as *bzip19/23-OEMtFbZIP1*. For *MtFbZIP2*, transgenic seeds of three independently transformed lines (T2 generation exhibiting a 3:1 segregation ratio) were selected. The lines were referred to as *bzip19/23-pZIP4:GUS-OEMtFbZIP2.*

### Plant Material and Growth Conditions

The Arabidopsis genotypes used in this study were the wild-type accession Columbia (Col-0), the double T-DNA insertion mutant *bzip19 bzip23* (*bzip19/23*) obtained from a cross between homozygous *bzip19-1* (SALK_144252) and *bzip23-1* (SALK_045200) lines in the Col-0 background, as described previously (Assunção et al., 2010); the *bzip19/23*-OE19 line, an overexpression of *AtbZIP19* in the *bzip19/23* mutant, as described previously (Lilay et al., 2019); the *pZIP4:GUS* reporter line containing the promoter of Arabidopsis *ZIP4* transporter gene fused to GUS and the *bzip19/23-pZIP4:GUS* reporter line obtained from a cross between *bzip19/23* and *pZIP4:GUS* lines, as described previously (Castro et al., 2017). The *bzip19/23-OEMtFbZIP1* and *bzip19/23-pZIP4:GUS-OEMtFbZIP2* lines were described above. The *M. truncatula* R108 ecotype was used as wild-type.

For the analysis of *M. truncatula* plants, seeds were scarified with 96% H_2_SO_4_ for 5 min, rinsing five times with cold sterile ddH_2_O, superficial sterilization with 30% (v/v) commercial bleach for 7 min, and rinsing five times with sterile ddH_2_O. The seeds were germinated on filter paper soaked with sterile ddH_2_O in a Petri dish, placed at 4°C for 1–2 h, then maintained in the dark at 4°C for 3 days, followed by room temperature for 1 day, and then placed in the climate chamber to allow cotyledons to emerge. The germinated seedlings were transferred to pots filled with sand, in a total of four pots per Zn treatment, with 1 seedling per pot. They were watered with 1 L per week for 6 weeks with a modified half-strength Hoagland nutrient solution containing:1 mM Ca(NO_3_)_2_, 1 mM MgSO_4_, 2 mM KNO_3_, 1 mM NH_4_NO_3_, 1 mM KH_2_PO_4_, 50 μM Fe-Na-EDTA, 25 μM H_3_BO_3_, 3 μM MnSO_4_, 0.1 μM CuSO_4_, 0.5 μM (NH_4_)_6_Mo_7_O_24_, 50 μM KCl, buffered with 1 mM MES at pH 5.8, containing 2 μM ZnSO_4_ (Zn sufficiency; control) or 0.002 μM ZnSO_4_ (Zn deficiency; -Zn). For agar-grown Arabidopsis seedlings, sterilized seeds were sown on half-strength Murashige and Skoog (1/2 MS) medium containing 15 μM ZnSO_4_ (Zn sufficiency; control), or no added Zn (Zn deficiency; –Zn) as previously described (Lilay et al., 2019). Five seedlings for each genotype were sown on each plate, with control or –Zn 1/2 MS media, and grown for 14 days. Three plates (replicates) per Zn treatment were grown simultaneously. For hydroponically grown Arabidopsis plants, sterilized seeds were germinated and grown for 6 weeks with a modified half-strength Hoagland nutrient solution with 2 μM ZnSO_4_ (Zn sufficiency; control) or with 0.002 μM ZnSO_4_ (Zn deficiency; -Zn), with six plants per genotype as previously described (Lilay et al., 2019). The pot experiment, the MS plate assay and the hydroponic set-up, were performed in a growth chamber with 125 mmol photons m^–2^ s^–1^ white light, 22/20°C light/dark temperature, 70% relative humidity, and 8/16 h (hydroponics) or 16/8 h (pot experiment and plate assay) light/dark cycle.

### Real-Time Quantitative RT–PCR Analysis

Shoots and roots of four-week-old *M. truncatula* plants, sand-grown in control or –Zn nutrient solution, were harvested separately. Three plants per Zn treatment were harvested and immediately frozen in liquid nitrogen. Shoot and root tissue were ground using a mortar and pestle in liquid nitrogen, and total RNA was extracted using the Direct-zol RNA Kit (Zymo Research). Fourteen-day-old Arabidopsis seedlings of the wild-type, *bzip19/23* mutant, *bzip19/23*-*OE19* and *bzip19/23*-*OEMtFbZIP1* lines, grown in control and –Zn MS medium, were harvested and immediately frozen in liquid nitrogen in pools of five seedlings per genotype, per plate and Zn treatment (control or –Zn). Three different plates per Zn treatment were grown simultaneously and considered as biological replicates. Three independently transformed T3 homozygous lines of *bzip19/23-OEMtFbZIP1* were analyzed. Seedlings were grinded with liquid nitrogen in a microtube, with the help of polypropylene pestles, and total RNA was extracted using the RNeasy Plant Mini Kit (Qiagen). *M. truncatula* and Arabidopsis RNA samples quantity and integrity were assessed, and cDNA synthesis was performed as previously described (Lilay et al., 2019). Primers for RT–qPCR (Supplementary Table 3) were designed using NCBI Primer-BLAST,^9^ and the primer amplification efficiency for each primer pair was between 1.9 and 2.1. RT–qPCR was performed with a LightCycler 96 Real-Time PCR System (Roche Diagnostics), using HOT FIREPol EvaGreen qPCR Mix (Solis BioDyne) in a 20 μL PCR mixture, as described previously (Lilay et al., 2019). The *M. truncatula* ubiquitin gene (*MtUBQ*, Medtr3g091400.1) and Arabidopsis *Actin-2* (*ACT2*, At3g18780) were used to normalize the gene expression analysis in *M. truncatula* and Arabidopsis, respectively. The calculated cycle threshold (Ct) value for each gene was normalized to the reference gene’s calculated Ct value. The relative transcript levels were expressed against the wild-type grown under control conditions, and calculated according to the 2^–ΔΔ^ ^CT^ method (Livak and Schmittgen, 2001).

The *in silico* analysis of expression of *MtFbZIP1* and *MtFbZIP2* genes was performed using MtExpress V2, the *M. truncatula* expression atlas^10^ (Carrere et al., 2021). Normalized expression values (Log2TMM) for each gene were retrieved from three datasets (reference_dataset; abiotic_factor; biotic_stress). Expression was visualized as an XY scatter plot of the RNA-seq normalized expression value for each gene, i.e., MtFbZIP1 (MtrunA17_Chr4g0036971) versus MtFbZIP2 (MtrunA17_Chr3g0090531).

### Histochemical Staining for GUS Assay and Imaging

Histochemical GUS (ß-glucuronidase) staining was performed with 12-day-old seedlings of *pZIP4:GUS, bzip19/23-pZIP4:GUS, bzip19/23-pZIP4:GUS-OEAtbZIP19* and *bzip19/23-pZIP4:GUS-OEMtFbZIP2*, grown with control or –Zn MS medium, as described by Lilay et al. (2021). Three independently transformed T2 generation seedlings were tested. Seedlings were immersed in GUS staining solution containing 50 mM phosphate buffer, 10 mM Na_2_-EDTA, 20% (v/v) methanol, 0.1% (v/v) Triton X-100, 1.4 mM K_3_[Fe(CN)_6_], 1.4 mM K_4_[Fe(CN)_6_]⋅3H_2_O with 1.9 mM X-Gluc and were incubated overnight at 37°C in the dark (Jefferson et al., 1987). After incubation, the pigments were removed by successive incubations in 50%, 70% and 96% (v/v) ethanol, and the seedlings were stored in 50% (v/v) glycerol. Bright-field images of GUS-stained seedlings, were recorded using a Leica M205FA stereo fluorescence microscope equipped with a digital Leica DFC450 C camera.

### Subcellular Localization Analysis

Fourteen-day-old seedlings of the *bzip19/23-OEMtFbZIP1* and *bzip19/23-pZIP4:GUS-OEMtFbZIP2* lines grown in control or –Zn MS medium were analyzed with confocal laser scanning microscopy (CLSM). Roots were incubated in 50 μM propidium iodide (PI) solution for 30 s to stain cell walls. Roots were visualized using a Leica TCS SP5 II laser scanning confocal microscope (Leica Microsystems) with a HC PL APO CS × 63/1.30 Glycerine objective. Argon 458 and 514 nm laser lines were used for CFP and PI excitation, respectively. The emission settings were between 470 and 533 nm for CFP and between 602 and 684 nm for PI. Three independently transformed lines were analyzed, with observations of 2–3 seedlings per line and Zn treatment.

### Tissue Element Analysis

Shoots and roots from 6-week-old *M. truncatula* plants, sand-grown with control or –Zn nutrient solution (3–4 plants per Zn treatment), were harvested. Shoots and roots from 6-week-old hydroponically grown Arabidopsis plants from the wild-type, *bzip19/23*, *bzip19/23-OE19* and *bzip19/23*-*OEMtFbZIP1* lines (3–6 plants per line per Zn treatment), grown with control and –Zn nutrient solution, were harvested. For the *bzip19/23* plants grown at –Zn, six plants were harvested, following the same procedure, and were analyzed in pools of two. Shoot and root harvest, tissue digestion, multielemental analysis performed with Inductively Coupled Plasma Optical Emission Spectroscopy (ICP-OES), and data analysis performed with Agilent ICP Expert Software v.7.3, were previously described (Lilay et al., 2021).

### Statistical Analysis

To compare different lines and treatments, one-way ANOVA followed by Tukey’s *post hoc* test was performed with IBM SPSS Statistics V22.0, and the Student’s t-test was carried out in Microsoft Excel.

## Results

### Phylogenetic Analysis of F-bZIP Homologs From Legume Species

**FIGURE 1:**
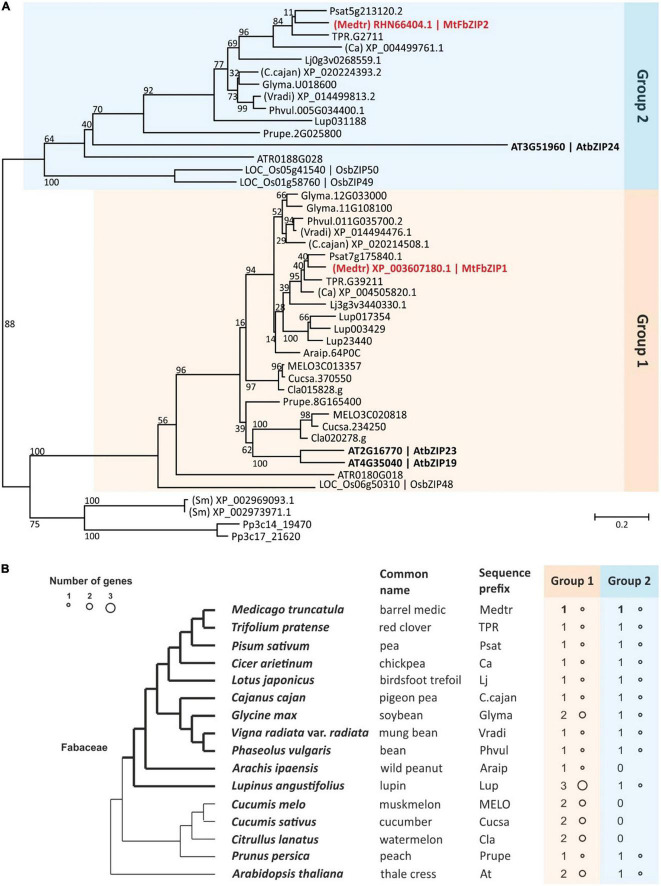
Phylogenetic analysis of the F-bZIP proteins in Fabaceae species. **(A)** Phylogenetic tree representing 11 species from the Fabaceae family, four species from the clade Fabids but not from the Fabaceae family (*Cucumis melo*, *Cucumis sativus*, *Citrullus lanatus*, and *Prunus persica*), and five representative species from major plant taxa (*Physcomitrella patens* (Pp), *Selaginella moellendorffii* (Sm), *Amborella trichopoda* (ATR), *Oryza sativa* (LOC_OS), and *Arabidopsis thaliana*). The species prefix of the 11 Fabaceae species, the four non-Fabaceae Fabid species and the five species representing major taxa are indicated here (above) and in panel **(B)**. The phylogenetic tree was constructed using maximum-likehood and shows bootstrap support from 1,000 replicates (numbers on each branch represent the percentages of bootstrap). **(B)** F-bZIP gene enrichment for the species from the Fabaceae family, non-Fabaceae Fabids and *A. thaliana* analyzed in the phylogeny. Circle size represents the number of genes for each species in Group 1 and Group 2.

A phylogenetic characterization of F-bZIP homologs across land plants had previously identified the presence of one F-bZIP member from *M. truncatula* (Castro et al., 2017). In order to obtain a more detailed evolutionary perspective of F-bZIP homologs from legume species, we performed a phylogenetic analysis with a set of 11 species from the Fabaceae family, i.e., *Arachis ipaensis, Cajanus cajan, Cicer arietinum, Glycine max, Lotus japonica, Lupinus angustifolius, M. truncatula*, *Phaseolus vulgaris, Pisum sativum, Trifolium pratense* and *Vigna radiata*. For evolutionary contextualization, the analysis included other species that served as representatives of major plant taxa (*P. patens*, *S. moellendorffii*, *A. trichopoda, O.sativa*, and *A. thaliana*) and species from the clade Fabids but not from the Fabaceae family (*P. persica*, *C. melo*, *C. sativus*, and *C. lanatus*), as described in the Methods section. In total, 24 F-bZIP sequences from 11 species of the Fabaceae family were identified and the full protein sequences were subsequently used for phylogenetic inference (Figure 1A). The sequences used in the phylogenetic analysis contain the conserved bZIP domain (basic region leucine-zipper) (Vinson et al., 2006) and the cysteine/histidine (Cys/His)-rich motif that is characteristic of F-bZIP proteins (Castro et al., 2017; Supplementary Table 1). The divergence of F-bZIP proteins into two groups, well established in previous phylogenetic analysis of F-bZIP homologs across land plants (Castro et al., 2017), and phylogenetic analysis of F-bZIP homologs enriched with Monocot species (Lilay et al., 2020), was also observed in this analysis. More specifically, the Bryophyte and Pteridophyte sequences’ positioning supports a single monophyletic origin for F-bZIP proteins, with differentiated branches forming Group 1 and Group 2 F-bZIPs (Figure 1A). In line with the previously reported phylogenetic analysis by Castro et al. (2017), we identified one Group 1 F-bZIP member for *M. truncatula*. Its gene ID is Medtr4g073100 based on Plaza Dicots 4.5 and Phytozome, which uses the *M. truncatula* genome assembly MedtrA17_4.0. Considering the recent release of a newer *M. truncatula* genome assembly (MedtrA17_5.0^11^), we carried out a protein blast in NCBI against the *M. truncatula* A17 r5.0 genome, and were able to detect a second F-bZIP gene (MtrunA17_Chr3g0090531). In this newer genome assembly, MedtrA17_5.0, the gene ID of Medtr4g073100 is MtrunA17_Chr4g0036971. To disambiguate the identification of the second *M. truncatula* F-bZIP, we analyzed the locus’ positioning in the genome and confirmed that it belongs to a small genomic region that was not present in the previous *M. truncatula* MedtrA17_4.0 assembly. Importantly, this second F-bZIP mapped as a Group 2 F-bZIP in our phylogenetic analysis (Figure 1A). The *M. truncatula* F-bZIPs MtrunA17_Chr4g0036971 and MtrunA17_Chr3g0090531 were annotated as MtFbZIP1 and MtFbZIP2, respectively. The gene enrichment plot deduced from the phylogenetic analysis (Figure 1B) shows that all analyzed species have at least one F-bZIP member in Group 1. All Fabaceae family members possess one F-bZIP member from Group 2, with the exception of *Arachis ipaensis*, while in the sister clade Cucurbitaceae, the loss of Group 2 members is observed. The *Arachis ipaensis* exception may derive from poor genome assembly/gene curation, and it would seem that the consensus is the presence of one Group 1 and one Group 2 F-bZIP members across the Fabaceae family.

### *M. truncatula F-bZIPs* Are Not Transcriptionally Responsive to Zn Deficiency

**FIGURE 2:**
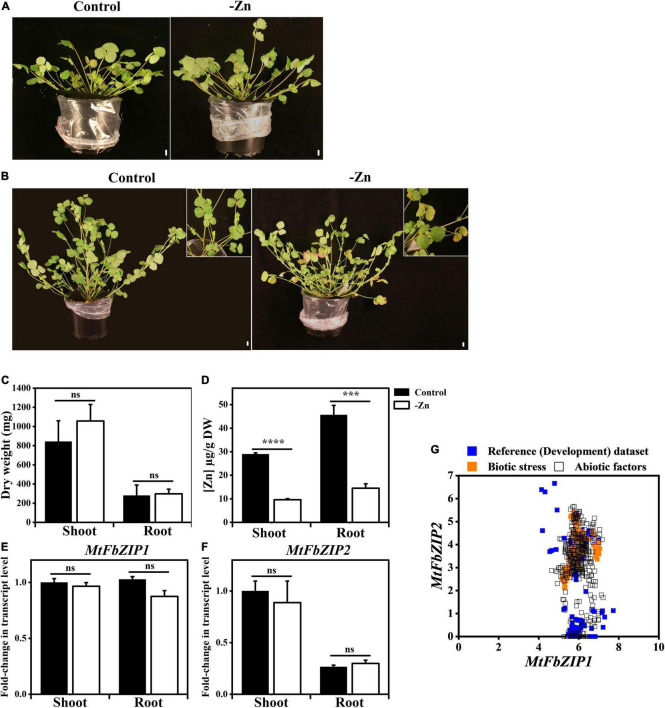
Analysis of *M. truncatula* plants exposed to Zn deficiency. Phenotypes of **(A)** 4-week-old and **(B)** 6-week-old *M. truncatula* plants grown on sand with nutrient solution at Zn sufficiency (Control) or Zn deficiency (–Zn). Top-right inset in panel **(B)** shows a detail of plant leaves. Scale bar = 1 cm. Quantification of **(C)** shoot and root dry weight and **(D)** shoot and root Zn concentration in 6-week-old plants. **(E–G)** Expression analysis of *M. truncatula F-bZIP* genes. Shoot and root transcript levels of **(E)** *MtFbZIP1* and **(F)** *MtFbZIP2* in 4-week-old plants grown at Zn sufficiency (Control) or Zn deficiency (–Zn). Data are represented as means ± SE (*n* ≥ 3). Significant differences between Control and –Zn treatments were determined by Student’s *t*-test (***p < 0.001, ****p < 0.0001, ns means not significant). **(G)** Comparative gene expression atlas analysis between *MtFbZIP1* (x-axis) and *MtFbZIP2* (y-axis) expression values using RNA-seq datasets from *M. truncatula* MtExpress (https://medicago.toulouse.inrae.fr/MtExpress). Log2/TMM (scale) is used for absolute normalized gene expression values. The different RNA-seq experimental conditions are grouped into three categories: reference (development), abiotic stress and biotic stress dataset (Supplementary Figure 2).

To investigate the role of the identified *M. truncatula* F-bZIP proteins (Figure 1) in the Zn deficiency response, we performed gene expression analysis in *M. truncatula* plants grown under Zn sufficient or Zn deficient conditions. Four-week-old plants, grown in sand and supplied with nutrient solution, showed mild phenotypic differences between the two Zn supply conditions (Figure 2A), whereas in the six-week-old plants the Zn deficiency treatment showed symptoms of leaf chlorosis (Figure 2B). Although there were no significant differences between Zn treatments regarding plant shoot or root dry weight (Figure 2C), the element analysis showed a significant reduction in the concentration of Zn in shoots and roots of plants grown at Zn deficiency, in comparison to Zn sufficiency (Figure 2D). The analysis of other nutrient elements (Fe, Cu, Mn and P) did not show significant differences between Zn supply conditions, except for Fe concentration in the shoot, which was significantly lower at Zn sufficiency than Zn deficiency (Supplementary Figure 1).

Next, we analyzed the expression of *MtFbZIP1* and *MtFbZIP2* genes. The transcript levels of *MtFbZIP1* and *MtFbZIP2* were not significantly different between plants grown with Zn sufficiency and Zn deficiency, both in shoots and roots (Figures 2E,F). Results also indicate that *MtFbZIP1* has a similar expression level in shoots and roots. In order to obtain information on the comparative expression between the two genes, we looked at available RNA-seq datasets from the *M. truncatula* RNA-seq Gene Expression Atlas Project (see text footnote 11). Expression data, comprising development, abiotic and biotic stress datasets, were retrieved for each gene and plotted in XY scatter for comparison of the gene pair’s expression values in the same experimental samples (Figure 2G and Supplementary Figure 2). The results suggest that the *MtFbZIP1* gene is consistently expressed across all tissues and stress conditions, within the expression value range of 4 to 8. This strongly points toward *MtFbZIP1* having a constitutive expression. On the other hand, the expression of the *MtFbZIP2* gene varies extensively with tissue type and abiotic stress imposition, showing relatively consistent expression levels within the biotic stress dataset (Figure 2G).

### Functional Analysis of MtFbZIP1 Indicates a Role in the Zn Deficiency Response

**FIGURE 3:**
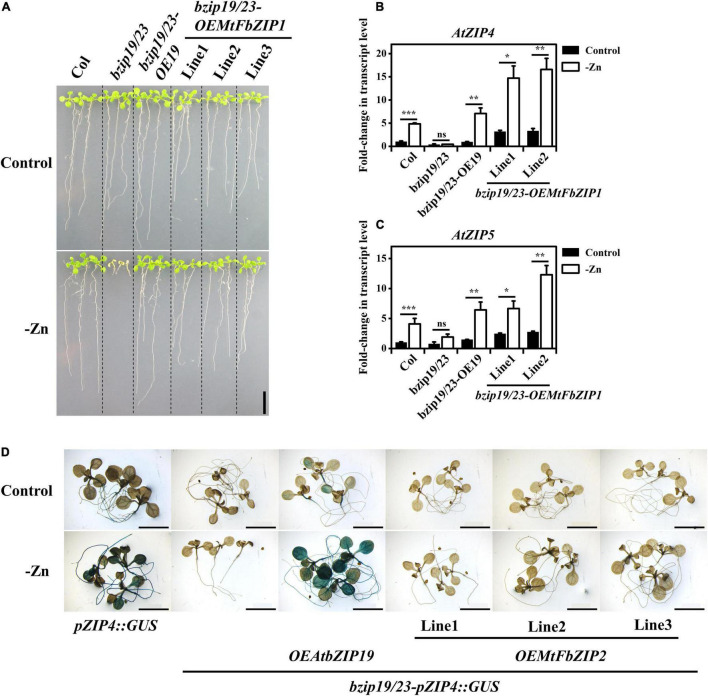
Functional analysis of *MtFbZIPs* in Arabidopsis *bzip19/23* double mutant. **(A–C)** Complementation analysis with *bzip19/23-OEMtFbZIP1* lines, wild-type (Col), *bzip19/23* double mutant and *bzip19/23*-*OEAtbZIP19* (*bzip19/23*-*OE19*; Lilay et al., 2019) grown at Zn sufficiency (Control) or Zn deficiency (–Zn). Homozygous T3 progeny of three independently transformed lines of *bzip19/23-OEMtFbZIP1* are designated Line 1–3. **(A)** Phenotype of 2-week-old seedlings grown on MS medium. Scale bar = 1 cm. **(B,C)** Gene expression analysis of **(B)** Arabidopsis *ZIP4* (*AtZIP4*) and **(C)** Arabidopsis *ZIP5* (*AtZIP5*) in 2-week-old seedlings. Data represent mean-fold change in transcript level ± SE (*n* = 2–3 biological replicates). Significant differences between Control and –Zn treatments were determined by Student’s *t*-test (*p < 0.05, **p < 0.01, ***p < 0.001, ns means not significant). **(D)** Histochemical GUS staining analysis of *pZIP4:GUS*, *bzip19/23-pZIP4:GUS*, *bzip19/23-pZIP4:GUS-OEAtbZIP19 (bzip19/23-pZIP4:GUS-bZIP19;* Lilay et al., 2021) and *bzip19/23-pZIP4:GUS-OEMtFbZIP2* lines. The images represent 10–15 12-day-old seedlings grown with Zn sufficient (Control) or Zn deficient (–Zn) MS medium. The *bzip19/23-pZIP4:GUS-OEMtFbZIP2* lines are T2 progeny of three independently transformed lines designated Line 1–3. Scale bars = 5 mm.

Expression analysis of the *MtFbZIP* genes indicated that *MtFbZIP1* is generally more expressed than *MtFbZIP2* (Figure 2G), and considering that MtFbZIP1 maps to Group 1 F-bZIPs (Figure 1A) as the Arabidopsis bZIP19 and bZIP23 (Castro et al., 2017), we started with the functional analysis of MtFbZIP1. To that effect, we performed a heterologous complementation analysis by expressing *MtFbZIP1* in the Arabidopsis *bzip19/23* background, which is characterized by a Zn deficiency hypersensitive phenotype (Assunção et al., 2010). To generate the complementation lines, the CDS of *MtFbZIP1* under control of the constitutive CaMV 35S promoter and containing a C-terminal CFP-HA fusion was stably transformed into the *bzip19/23* double mutant (i.e., *bzip19/23-OEMtbFZIP1*). The transcript levels of *MtFbZIP1* in three independently transformed lines were verified (Supplementary Figure 3). To test the complementation of the *bzip19/*23 Zn deficiency phenotype, the *bzip19/23-OEMtFbZIP1* lines were grown on a plate assay. The analysis of 14-day-old seedlings at Zn deficiency showed that the lines overexpressing *MtFbZIP1* were able to complement the *bzip19/23* mutant phenotype (Figure 3A). At Zn deficiency, the growth of the seedlings was comparable between the *bzip19/23-OEMtFbZIP1* lines and the wild-type, whereas the *bzip19/23* line exhibited reduced growth and chlorosis. The *bzip19/23* mutant is fully complemented with the Arabidopsis bZIP19 transcription factor (*bzip19/23-OE19*) (Lilay et al., 2019), and here it was used as an additional control. At Zn sufficiency, there was no visible difference between the seedlings from all analyzed lines (Figure 3A). Seedlings from the plate assay were further analyzed for the expression of two Arabidopsis *ZIP* genes (*AtZIP4* and At*ZIP5*), which are targets of AtbZIP19 and AtbZIP23 transcription factors (Assunção et al., 2010). The transcript levels of *AtZIP4* and *AtZIP5* were higher at Zn deficiency than Zn sufficiency in the wild-type, *bzip19/23-OE19* and *bzip19/23-OEMtFbZIP1* lines, but not in the *bzip19/23* mutant, which had low transcript level at both Zn conditions (Figures 3B,C). Although the transcript level of *AtZIP4 and AtZIP5* in the *bzip19/23-OEMtFbZIP1* was generally higher than in the wild-type and *bzip19/23-OE19*, they all showed a comparable pattern of response to Zn supply (Figures 3B,C). We also looked at the *bzip19/23-OEMtFbZIP1* lines grown in hydroponics, in a longer-term experiment. The 5-week-old plants grown at Zn deficiency showed a partial complementation of the Zn deficiency phenotype in comparison with the wild-type, whereas under control conditions there were no visible differences between plants, in line with the shoot dry weight and tissue Zn concentration data (Supplementary Figure 4). This partial complementation in plants could relate to the fact that it is a heterologous complementation with a *M. truncatula* gene in Arabidopsis background. Overall, the functional complementation analysis, including the expression of AtbZIP19/23 target genes, clearly indicate that MtFbZIP1 protein is a functional homolog of Arabidopsis bZIP19 and bZIP23 transcription factors.

### MtFbZIP2 Does Not Activate the *pZIP4:GUS* Reporter

We proceeded to obtain functional information on the MtFbZIP2. To that effect we used the reporter line *pZIP4:GUS*, in Col-0 wild-type background, which uses the target gene *AtZIP4* as a Zn deficiency marker. While the *bzip19/23-pZIP4:GUS* line does not show GUS reporter activity (Figure 3D) (Castro et al., 2017), the Zn-dependent activity of *pZIP4:GUS* reporter is restored upon complementation with the *AtbZIP19 (bzip19/23-pZIP4:GUS-OEAtbZIP19*) (Figure 3D) (Lilay et al., 2021). Here, we tested this reporter system with the *MtFbZIP2*. To generate the complementation lines, the CDS of *MtFbZIP2* under control of the constitutive CaMV 35S promoter and containing a C-terminal CFP-HA fusion was stably transformed into the *bzip19/23-pZIP4:GUS* line (i.e., *bzip19/23-pZIP4:GUS-OEMtbFZIP2*). The analysis of 12-day-old seedlings in a plate assay showed that the lines overexpressing *MtFbZIP2* did not show GUS expression, likewise the *bzip19/23-pZIP4:GUS* line (Figure 3D). The *pZIP4:GUS* expression patterns in the wild-type background (*pZIP4:GUS*), in the *bzip19/23* mutant (*bzip19/23-pZIP4:GUS*) and in the complementation line with *AtbZIP19* (*bzip19/23-pZIP4:GUS-OEAtFbZIP19*) (Figure 3D) are in agreement with the transcript levels of the *AtZIP4* gene in the wild-type, *bzip19/23* and *bzip19/23*-OE19 lines, respectively (Figure 3B). The *bzip19/23* lines complemented with *MtFbZIP1* show Zn deficiency induced *AtZIP4* gene expression, whereas the *bzip19/23-pZIP4:GUS* lines complemented with *MtFbZIP2* show no Zn deficiency induced *pZIP4:GUS* expression (Figures 3B,D). These results reveal that the MtFbZIP2 does not activate AtbZIP19/23 target gene expression, indicating that MtFbZIP2 is not a functional homolog of AtbZIP19/23 transcription factors and does not play a role in the Zn deficiency response.

### MtFbZIP1 and MtFbZIP2 Localize in the Nucleus

**FIGURE 4:**
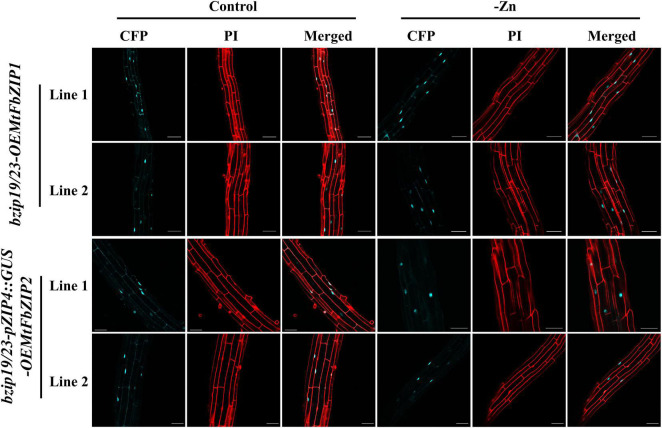
Subcellular localization analysis of MtFbZIP1-CFP and MtFbZIP2-CFP fusion proteins in Arabidopsis roots. Two-week-old seedlings of *bzip19/23-OEMtFbZIP1* and *bzip19/23-pZIP4:GUS-OEMtFbZIP2* lines, grown on MS media at Zn sufficiency (Control) or Zn deficiency (–Zn) were analyzed. Emissions of CFP and propidium iodide (PI) were determined by CLSM. Two to three seedlings for each independent line were used. Scale bars = 50 μm.

To investigate the subcellular localization of the MtFbZIP1 and MtFbZIP2 proteins, we analyzed seedling roots of the *bzip19/23-OEMtFbZIP1* and *bzip19/23-pZIP4:GUS-OEMtFbZIP2* lines, grown at Zn sufficiency and Zn deficiency. The fluorescence signal of the C-terminal Cyan Fluorescent Protein (CFP) fluorophore of the MtFbZIP1 and MtFbZIP2 fusions (MtFbZIP1-CFP and MtFbZIP2-CFP, respectively) were visualized with confocal laser scanning microscopy (CLSM). The analyses showed that the MtFbZIP1 and MtFbZIP2 fusion proteins localized in the nucleus of the cell. The analysis of seedlings grown at Zn deficiency also revealed that the subcellular localization was identical between seedlings grown at Zn sufficient or Zn deficient conditions. This indicates that the cellular Zn status is not involved in the subcellular targeting of *M. truncatula* F-bZIP proteins (Figure 4).

### Analysis of *M. truncatula ZIP* and *NAS* Genes as Candidate Targets of MtFbZIP1

**FIGURE 5:**
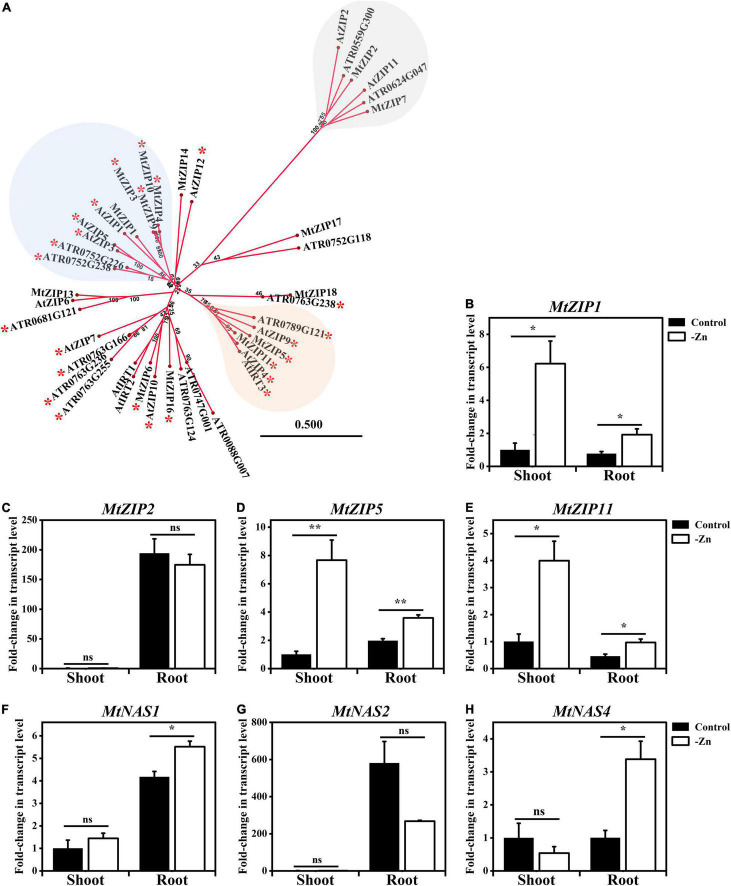
Analysis of *M. truncatula* ZIP and NAS family members. **(A)** Phylogenetic tree of *M. truncatula* (Mt), Arabidopsis (At) and *A. trichopoda* (ATR) ZIP transporters. Branch lengths are proportional to phylogenetic distance. Numbers on each branch represent the percentages of bootstrap. An asterisk (*) next to a ZIP transporter gene ID means that one or more ZDRE motifs were detected in the gene promoter (Supplementary Table 2). The orange, blue and gray shadows highlight different clades of the phylogenetic tree. **(B–H)** Gene expression analysis of 4-week-old *M. truncatula* plants grown on sand watered with Zn sufficient (Control) or Zn deficient (–Zn) nutrient solution. Shoot and root transcript level of **(B)** *MtZIP1*, **(C)** *MtZIP2*, **(D)** *MtZIP5*, **(E)** *MtZIP11*, **(F)** *MtNAS1*, **(G)** *MtNAS2*, and **(H)** *MtNAS4*. Data represent mean fold-change in transcript level ± SE (n = 3 biological replicates). Significant differences between control and –Zn treatments were determined by Student’s *t*-test (*p < 0.05, **p < 0.01, ns means not significant).

To examine the conservation of the Zn deficiency response in *M. truncatula*, and in order to identify candidate target genes of MtFbZIP1, we analyzed the *M. truncatula* genes encoding ZIP family transporters and NAS enzymes. First, to visualize the relationship between *M. truncatula* and Arabidopsis ZIP transporters, we produced a phylogenetic tree, including 15 *M. truncatula* and 14 Arabidopsis ZIP members. In addition, 14 ZIP members from *Amborella trichopoda* were included, representing a basal lineage in the clade of Angiospersms, and in order to provide evolutionary context (Figure 5A). The tree showed the presence of ZIP proteins from the three species in most of the clades produced in the phylogeny, thus resolving distinct functional ortholog groups within the different ZIP homologs. As an example, the clade with the Arabidopsis ZIP4/9 and IRT3 transporters suggests that MtZIP5 and MtZIP11 are their functional equivalents in *M. truncatula*. Overall results are in line with previous phylogenetic analysis of Arabidopsis and *M. truncatula* ZIP proteins (Mäser et al., 2001; Abreu et al., 2017). Next, we analyzed the promoter region of all *ZIP* genes used in the phylogenetic analysis, to search the presence of ZDRE cis-regulatory elements to which the Arabidopsis bZIP19 and bZIP23 transcription factors bind to (Assunção et al., 2010). The consensus sequence RTGWCGACAY was used, and an overview of the detected ZDRE motifs, including their numbers and positions, is shown in Supplementary Table 2. In general, ZDREs were identified in the promoters of *M. truncatula* and *A. trichopoda ZIP* genes which are in clades together with Arabidopsis *ZIP* genes that are targets of AtbZIP19/23. Accordingly, no ZDRE motifs were detected in the promoters of *M. truncatula* and *A. trichopoda ZIP* genes positioned in the clade containing the Arabidopsis *ZIP2*, which is not a target gene of AtbZIP19/23 (Figure 5A). In order to extend the information on the presence of ZDRE promoter elements to the *M. truncatula NAS* genes, a similar *in silico* search was performed in the four annotated *MtNAS* genes (Supplementary Table 2).

Based on the phylogeny of ZIP transporters, we focused on four *MtZIPs* (*MtZIP1/2/5/11*) to investigate their gene expression in *M. truncatula* plants in response to Zn supply. As mentioned, MtZIP5 and MtZIP11 are close homologs of AtZIP4/9 and IRT3 (Figure 5A, orange shadow). In a different clade, MtZIP1 is a close homolog of AtZIP1 (Figure 5A, blue shadow). All the Arabidopsis ZIP members from these two clades are target genes of AtbZIP19/23. In a more distantly related clade, MtZIP2 is a close homolog of AtZIP2, (Figure 5A, gray shadow) a non-target gene. The expression analysis showed that the transcript levels of *MtZIP1*, *MtZIP5* and *MtZIP11* were significantly higher in Zn-deficient than in Zn-sufficient plants. The three genes had higher Zn-deficiency induced expression in shoots than in roots (Figures 5B,D,E). The transcript level of *MtZIP2* was not significantly different between the Zn treatments, and *MtZIP2* was more expressed in roots, with a comparatively very low detection in shoots (Figure 5C). The gene expression analysis was extended to the four *MtNAS* genes (*MtNAS1-4*). The transcript levels of *MtNAS1* and *MtNAS4* were induced in Zn-deficiency in roots but not in shoots (Figures 5F,H). The transcript level of *MtNAS2* was not significantly affected by Zn treatments, with *MtNAS2* more expressed in roots than in shoots (Figure 5G). For *MtNAS3*, we could not detect transcript level signal. Concerning the presence of promoter ZDRE motifs in these genes, we identified the presence of two motifs in both *MtZIP5* and *MtZIP11* promoters, with one mismatch in one ZDRE from *MtZIP5*. In addition, we identified one ZDRE in both, the *MtNAS1* and in *MtNAS4* promoters, with one mismatch in *MtNAS1* (Supplementary Table 2). Together these results suggest an association between the presence of ZDREs in the promoter of *M. truncatula ZIP* and *NAS* genes and their transcriptional response to Zn deficiency.

## Discussion

### Phylogenetic Analysis Identifies Two *M. truncatula* F-bZIP Homologs

In order to obtain a more detailed evolutionary characterization of F-bZIP homologs from legume species, we performed a phylogenetic analysis enriched in species from the Fabaceae family. The analysis supported the emergence of F-bZIP Groups 1 and 2 associated with seed plant differentiation (Figure 1). This is in line with our previous phylogenetic analysis of F-bZIP homologs across land plants (Castro et al., 2017), which was further supported in a phylogenetic and synteny analysis enriched in Monocot species (Lilay et al., 2020). Here, we identified two *M. truncatula* F-bZIP homologs: MtFbZIP1 (MtrunA17_Chr4g0036971) in Group 1 and MtFbZIP2 (MtrunA17_Chr3g0090531) in Group 2. In the previous phylogenies, all species had at least one Group 1 F-bZIP, whereas Group 2 appeared more prone to gene loss or expansion events (Castro et al., 2017). For example, indication of gene expansion in Group 2 was observed in the Monocot enriched analysis (Lilay et al., 2020). In this phylogeny, a Group 1 F-bZIP is also present in all species, whereas Group 2, rather than expansion or loss, displays conservation of a single member per Fabaceae genome, while in the sister clade Cucurbitaceae the loss of Group 2 members is observed. Thus, this phylogenetic analysis reinforces the branching of F-bZIPs into two groups, with F-bZIP homologs from Group 1 consistently present in all species, with at least one member, whereas homologs from Group 2 display more variable evolutionary paths. The central regulators of the Zn deficiency response in Arabidopsis, AtbZIP19 and AtbZIP23, and its functional homolog in rice, OsbZIP48, belong to Group 1 (Assunção et al., 2010; Lilay et al., 2020). The Arabidopsis F-bZIP Group 2 single member, AtbZIP24, has no major role in the Zn deficiency response (Lilay et al., 2019), and is involved in salt stress regulation (Yang et al., 2009). In rice, bZIP49 from Group 2 seems to be a truncated protein not involved in the Zn deficiency response. Whereas OsbZIP50, also from Group 2, plays a role in the Zn deficiency response, however its ectopic expression in Arabidopsis suggests an altered regulatory response at Zn sufficiency (Lilay et al., 2020). These observations support the suggested conservation of the Zn deficiency response associated with Group 1 members, while the Group 2 F-bZIPs, on the other hand, are more prone to gene loss or expansion events that might lead to non-, sub- or neo-functionalization (Castro et al., 2017). To obtain information about the *M. truncatula* F-bZIPs, we proceeded with their functional characterization.

### MtFbZIP1 Is the Functional Homolog of Arabidopsis bZIP19/23 Transcription Factors

To investigate the role of *M. truncatula* F-bZIPs in the Zn deficiency response, we analyzed *M. truncatula* plants grown with different Zn supply (Figure 2). At Zn deficiency, plants showed a mild visible phenotype, but the element analysis in shoot and root showed a reduced Zn concentration in comparison to the control condition. The expression of *MtFbZIP1* and *MtFbZIP2* genes in these plants was not Zn-responsive, indicating that they are not transcriptionally regulated by Zn status. Similar results were obtained for Arabidopsis *F-bZIP* genes *AtbZIP19/23/24* (Lilay et al., 2019) and for rice *F-bZIP* genes *OsbZIP48/49/50* (Lilay et al., 2020). Current knowledge in Arabidopsis indicates that the Zn-dependent activity of the AtbZIP19/23 is not controlled at the transcriptional level, but at the protein level where activity is repressed at Zn sufficiency (Lilay et al., 2019). This is the result of a direct binding of Zn^2+^ ions to the characteristic Cys/His-rich motif, the Zn-sensor motif (ZSM), of AtbZIP19/23 transcription factors, which also act as direct sensors of the intracellular Zn concentration (Lilay et al., 2021). In order to obtain information on the comparative expression between the *MtFbZIP1* and *MtFbZIP2* genes, we analyzed available RNA-seq datasets from *M. truncatula* (Figure 3). Expression data, comprising development, abiotic and biotic stress datasets, indicated that *MtFbZIP1* is broadly expressed with a more constitutive pattern across different tissue types and stress factors than *MtFbZIP2*, with the latter showing a high degree of variation.

Next, we performed a functional characterization of MtFbZIP1 by heterologous complementation analysis in the Arabidopsis *bzip19/23* double mutant. The *bzip19/23* mutant is hypersensitive to Zn deficiency, and overexpression of *AtbZIP19* or *AtbZIP23* functionally complements the mutant. In these complementation lines, the transcriptional activation of the Arabidopsis bZIP19/23 target genes at Zn deficiency is restored (Lilay et al., 2019). Here, the analysis of *bzip19/23-OEMtFbZIP1* seedlings showed complementation of the *bzip19/23* Zn deficiency phenotype, and the expression of Arabidopsis *ZIP* transporter target genes, *AtZIP4* and *AtZIP5*, was induced at Zn deficiency (Figure 4). Comparable results were observed in heterologous complementation analysis of wheat, barley and rice F-bZIPs in the Arabidopsis *bzip19/23* mutant (Evens et al., 2017; Nazri et al., 2017; Lilay et al., 2020). We also performed a functional analysis of MtFbZIP2 in the Arabidopsis *bzip19/23-pZIP4:GUS* line, which uses the *AtZIP4* promoter fused to GUS as a reporter for transcriptional activation by AtbZIP19/23 in response to Zn deficiency, as described in Lilay et al. (2021). However, GUS expression was not observed in the *bzip19/23-pZIP4:GUS-OEMtFbZIP2* seedlings. These results indicate that MtFbZIP1, but not MtFbZIP2, functionally complement the *bzip19/23* mutant. Localization analysis showed that both, MtFbZIP1 and MtFbZIP2, are in the nucleus and their nuclear localization seems to be independent of cellular Zn status. Comparable results were reported in localization analysis of Arabidopsis (AtbZIP19/23/24) and rice (OsbZIP48/49/50) F-bZIP proteins, from Group 1 and Group 2 (Lilay et al., 2019, 2020). Overall, results indicate that Group 1 MtFbZIP1 is a functional homolog of the Arabidopsis bZIP19 and bZIP23 transcription factors, and, as such, it is likely a regulator of the Zn deficiency response in *M. truncatula*. On the other hand, Group 2 MtFbZIP2 does not seem to play a role in the Zn deficiency response. The RNA-seq analysis showed *MtFbZIP2* gene expression varying extensively with tissue type and abiotic stress imposition, but more consistent within the biotic stress dataset (Figure 3), suggesting that MtFbZIP2 may be sub-functionalizing into specific roles. This is in line with the suggested conservation of the Zn deficiency response associated with Group 1 members, whereas Group 2 displays more variable evolutionary paths (Castro et al., 2017).

### Exploring the F-bZIP Regulated Zn Deficiency Response in *M. truncatula*

To further unravel the Zn deficiency response in *M. truncatula*, we investigated the putative MtFbZIP1 regulatory network, and analyzed the expression of *M. truncatula ZIP* and *NAS* genes in response to Zn supply. In Arabidopsis, bZIP19/23 target genes include members of the ZIP transporter family and NAS enzymes, which contain ZDRE motifs in the promoter and are transcriptionally activated at Zn deficiency (Assunção et al., 2010). The ZIP family members are divalent cation transporters involved in uptake of Zn, Fe and manganese (Mn) into the cell cytoplasm (Guerinot, 2000; Sinclair and Krämer, 2012). The *ZIP* target genes of AtbZIP19 and AtbZIP23 are *AtZIP1/3/4/5/9/10/12 and AtIRT3*, and *AtIRT3*, and except for *AtZIP5*, the encoded proteins have been shown to mediate Zn transport (Grotz et al., 1998; Lin et al., 2009; Assunção et al., 2010; Milner et al., 2013; Lee et al., 2021). In *M. truncatula*, there are 15 annotated members from the ZIP family, where MtZIP1-7 were characterized and shown to mediate Zn, Fe and Mn transport (Burleigh et al., 2003; López-Millán et al., 2004; Abreu et al., 2017). MtZIP6 is involved in Zn uptake by rhizobia-infected nodule cells, playing a role in the symbiosome Zn homeostasis (Abreu et al., 2017). To obtain information on the relationship between Arabidopsis and *M. truncatula* ZIP transporters, we produced a phylogenetic tree to which we included the detected ZDRE motifs in the *ZIP* gene promoters (Figure 5). Previously, the analysis of gene promoters of *AtZIP4/9* and *AtIRT3* homologs across land plants identified an enrichment in ZDREs in the promoter regions (Castro et al., 2017). Here, the phylogenetic analysis supports such enrichment, and additionally, it highlights an enrichment of ZDREs in other clades containing other *AtZIP* target genes of AtbZIP19/23. The *MtZIP1*, *MtZIP5* and *MtZIP11*, close homologs of *AtZIP* target genes, and on the other hand, *MtZIP2*, a close homolog of *AtZIP2*, a non-target gene, were analyzed in *M. truncatula* plants (Figure 5; Abreu et al., 2017). The expression of *MtZIP1*, *MtZIP5* and *MtZIP11* genes is induced by Zn deficiency, both in shoots and roots, but with higher transcript level in shoots than in roots. This is consistent with earlier observations on *MtZIP1* and *MtZIP5* expression, except that *MtZIP5* expression in roots was previously reported as non Zn-responsive (López-Millán et al., 2004). MtZIP5 and MtZIP11 were functionally characterized as Zn transporters (López-Millán et al., 2004), and, considering their Zn-responsive gene expression patterns and presence of ZDRE promoter elements (Figure 5), they constitute target gene candidates of the MtFbZIP1 transcription factor, to play a role in the Zn deficiency response. MtZIP1 also mediates Zn transport (López-Millán et al., 2004), but no ZDRE was detected in the promoter of *MtZIP1* gene. This may suggest regulation by transcription factors other than the F-bZIPs, or other F-bZIP-DNA binding *cis*-elements. The expression of *MtZIP2* gene is not Zn-deficiency responsive and it does not contain ZDREs in the promoter, which is in line with reports for *AtZIP2*, and for rice *OsZIP2*, another *AtZIP2* homolog (Assunção et al., 2010; Lilay et al., 2020). Previously, MtZIP2 was shown to mediates Zn transport, with *MtZIP2* mainly expressed in roots (Burleigh et al., 2003), in line with our observations. This supports a role for MtZIP2 in *M. truncatula* Zn homeostasis, but not in the F-bZIP mediated Zn deficiency response.

In addition, to *MtZIP* we also analyzed *MtNAS* genes. The NAS enzymes produce NA ligand that forms complexes with Zn, Fe and other metal micronutrients. NA is involved in Zn and Fe homeostasis and contributes to their intercellular and long-distance distribution (Clemens, 2019). In *M. truncatula*, MtNAS1 is suggested to play a role in the efficient Fe supply to the nodule with impact in nitrogen fixation acclimation efficiency (Avenhaus et al., 2016), while MtNAS2 is required for symbiotic nitrogen fixation (Escudero et al., 2020), supporting an important role for NA in Fe delivery and Fe homeostasis in the symbiosome. In Arabidopsis, *AtNAS2* and *AtNAS4* are among the target genes of AtbZIP19 and AtbZIP23, and are transcriptionally activated at Zn deficiency (Assunção et al., 2010). Here, the expression of *MtNAS2* gene was not induced by Zn deficiency and it is mainly expressed in roots, in agreement with Escudero et al. (2020), whereas the expression of *MtNAS1* and *MtNAS4* was induced by Zn deficiency in roots, but not in shoots. In the promoter of *MtNAS1* we found one ZDRE with one mismatch (Supplementary Table 2). However, its relevance is questionable since a mutation in the same position (i.e., RTGTAGACAY) was previously shown to fail binding to Arabidopsis AtbZIP19 and AtbZIP23, and to rice OsbZIP48, in pull-down *in vitro* assays (Assunção et al., 2010; Lilay et al., 2020) suggesting that it is an essential nucleotide for the F-bZIP-DNA binding. *MtNAS4* showed stronger Zn-deficiency induced expression than *MtNAS1* and it contains one ZDRE in the promoter, therefore *MtNAS4* could be a candidate target gene of MtFbZIP1. Nevertheless, the regulation of *NAS* genes seems to be complex and controlled by multiple mechanisms. In Arabidopsis, bHLH and MYB transcription factors, which regulate the Fe deficiency response, control the expression of *NAS* genes, with indication of cross-talk between Fe and Zn homeostasis (Palmer et al., 2013; Chen et al., 2018). Overall, the analysis of *M. truncatula ZIP* and *NAS* genes suggests that *MtZIP5*, *MtZIP11* and *MtNAS4* are candidate target genes of the MtFbZIP1 transcription factor, and integrate the Zn deficiency response regulatory network in *M. truncatula*.

## Concluding Remarks

Our results suggest that the Zn deficiency response regulated by F-bZIP transcription factors is conserved in the legume model *M. truncatula*. In legumes species, an important component of the plant Zn homeostasis is the transport of Zn to the nodule-rhizobia infected cells where an adequate delivery to metalloproteins is required for proper nitrogen fixation (Abreu et al., 2020). It is conceivable that the Zn deficiency response includes the regulation of Zn homeostasis in symbiosomes in *M. truncatula.* The functional analysis of the two identified *M. truncatula* F-bZIPs indicates that Group 1 MtFbZIP1 is the functional homolog of Arabidopsis bZIP19 and bZIP23 transcription factors. The analyses of *M. truncatula ZIP* and *NAS* genes, namely the Zn-deficiency responsive expression associated with the presence of ZDREs in the promoter region, support the conservation of the F-bZIP-regulated Zn deficiency response in *M. truncatula*. This conservation paves the way to follow-up investigations on the modulation of F-bZIP transcription factor’s activity to impact plant Zn content (Lilay et al., 2021). The analysis of Group 2 MtFbZIP2 suggests that it does not play a role in the Zn deficiency response and may be subfunctionalizing into other roles. A phylogenetic analysis of F-bZIP homologs enriched in legume species provides support for the translation of functional insight on the F-bZIP regulatory network from the model *M. truncatula* to legume crops as a strategy to obtain improved Zn nutritional value.

## Data Availability Statement

The original contributions presented in this study are included in the article/Supplementary Material, further inquiries can be directed to the corresponding author.

## Author Contributions

FL, GHL, and AGLA designed and performed the experimental work. FL and AGLA analyzed the data and wrote the manuscript. FL, PHC, and HA performed the phylogenetic analysis. All authors revised the manuscript.

## Conflict of Interest

The authors declare that the research was conducted in the absence of any commercial or financial relationships that could be construed as a potential conflict of interest.

## Publisher’s Note

All claims expressed in this article are solely those of the authors and do not necessarily represent those of their affiliated organizations, or those of the publisher, the editors and the reviewers. Any product that may be evaluated in this article, or claim that may be made by its manufacturer, is not guaranteed or endorsed by the publisher.

## Footnotes

1
https://bpi.harvestplus.org

2
https://bioinformatics.psb.ugent.be/plaza/versions/plaza_v4_5_dicots/

3
https://phytozome-next.jgi.doe.gov/

4
https://legumeinfo.org/

5
https://blast.ncbi.nlm.nih.gov/Blast.cgi?PAGE=Proteins

6
http://www.phylo.org

7
https://www.arabidopsis.org/

8
https://meme-suite.org/meme

9
www.ncbi.nlm.nih.gov/tools/primer-blast

10
https://medicago.toulouse.inrae.fr/MtExpress

11
https://medicago.toulouse.inra.fr/MtrunA17r5.0-ANR/

## References

[B1] Abreu I. Escudero V. Montiel J. Castro-Rodríguez R. González-Guerrero M. (2020). “Metal transport in *Medicago truncatula* nodule rhizobia-infected cells,” in *The Model Legume Medicago Truncatula*, ed. F de Bruijn (Hoboken: Wiley), 652–664. 10.1002/9781119409144.ch81

[B2] Abreu I. Saéz Á Castro-Rodríguez R. Escudero V. Rodríguez-Haas B. Senovilla M. (2017). Medicago truncatula Zinc-Iron Permease6 provides zinc to rhizobia-infected nodule cells. *Plant Cell Environ.* 40 2706–2719. 10.1111/pce.13035 28732146

[B3] Andreini C. Banci L. Bertini I. Rosato A. (2006). Zinc through the three domains of life. *J. Proteome Res.* 5 3173–3178. 10.1021/pr0603699 17081069

[B4] Assunção A. G. L. Herrero E. Lin Y.-F. Huettel B. Talukdar S. Smaczniak C. (2010). Arabidopsis thaliana transcription factors bZIP19 and bZIP23 regulate the adaptation to zinc deficiency. *Proc. Natl. Acad. Sci. U. S. A.* 107 10296–10301. 10.1073/pnas.1004788107 20479230 PMC2890486

[B5] Avenhaus U. Cabeza R. A. Liese R. Lingner A. Dittert K. Salinas-Riester G. (2016). Short-Term Molecular Acclimation Processes of Legume Nodules to Increased External Oxygen Concentration. *Front. Plant Sci.* 6:1133. 10.3389/fpls.2015.01133 26779207 PMC4702478

[B6] Beebe S. Gonzalez A. V. Rengifo J. (2000). Research on Trace Minerals in the Common Bean. *Food Nutr. Bull.* 21 387–391. 10.1177/156482650002100408

[B7] Burleigh S. H. Kristensen B. K. Bechmann I. E. (2003). A plasma membrane zinc transporter from Medicago truncatula is up-regulated in roots by Zn fertilization, yet down-regulated by arbuscular mycorrhizal colonization. *Plant Mol. Biol.* 52 1077–1088. 10.1023/a:1025479701246 14558666

[B8] Carrere S. Verdier J. Gamas P. (2021). MtExpress, a Comprehensive and Curated RNAseq-based Gene Expression Atlas for the Model Legume Medicago truncatula. *Plant Cell Physiol.* 62 1494–1500. 10.1093/pcp/pcab110 34245304

[B9] Castro P. H. Lilay G. H. Munoz-Merida A. Schjoerring J. K. Azevedo H. Assuncao A. G. L. (2017). Phylogenetic analysis of F-bZIP transcription factors indicates conservation of the zinc deficiency response across land plants. *Sci. Rep.* 7:3806. 10.1038/s41598-017-03903-6 28630437 PMC5476651

[B10] Castro-Guerrero N. A. Isidra-Arellano M. C. Mendoza-Cozatl D. G. Valdés-López O. (2016). Common Bean: A Legume Model on the Rise for Unraveling Responses and Adaptations to Iron, Zinc, and Phosphate Deficiencies. *Front. Plant Sci.* 7:600. 10.3389/fpls.2016.00600 27200068 PMC4853408

[B11] Champagne C. E. M. Goliber T. E. Wojciechowski M. F. Mei R. W. Townsley B. T. Wang K. (2007). Compound Leaf Development and Evolution in the Legumes. *Plant Cell* 19 3369–3378. 10.1105/tpc.107.052886 17993625 PMC2174894

[B12] Chen C.-L. Cui Y. Cui M. Zhou W.-J. Wu H.-L. Ling H.-Q. (2018). A FIT-binding protein is involved in modulating iron and zinc homeostasis in Arabidopsis. *Plant Cell Environ.* 41 1698–1714. 10.1111/pce.13321 29677391

[B13] Clemens S. (2001). Molecular mechanisms of plant metal tolerance and homeostasis. *Planta* 212 475–486. 10.1007/s004250000458 11525504

[B14] Clemens S. (2019). Metal ligands in micronutrient acquisition and homeostasis. *Plant Cell Environ.* 42 2902–2912. 10.1111/pce.13627 31350913

[B15] Clemens S. Deinlein U. Ahmadi H. Höreth S. Uraguchi S. (2013). Nicotianamine is a major player in plant Zn homeostasis. *BioMetals* 26 623–632. 10.1007/s10534-013-9643-1 23775667

[B16] Colvin R. A. Holmes W. R. Fontaine C. P. Maret W. (2010). Cytosolic zinc buffering and muffling: their role in intracellular zinc homeostasis. *Metallomics* 2 297–356. 10.1039/b926662c 21069178

[B17] Earley K. W. Haag J. R. Pontes O. Opper K. Juehne T. Song K. (2006). Gateway-compatible vectors for plant functional genomics and proteomics. *Plant J.* 45 616–629. 10.1111/j.1365-313X.2005.02617.x 16441352

[B18] Escudero V. Abreu I. del Sastre E. Tejada-Jiménez M. Larue C. Novoa-Aponte L. (2020). Nicotianamine Synthase 2 Is Required for Symbiotic Nitrogen Fixation in Medicago truncatula Nodules. *Front. Plant Sci.* 10:1780. 10.3389/fpls.2019.01780 32082345 PMC7003136

[B19] Evens N. P. Buchner P. Williams L. E. Hawkesford M. J. (2017). The role of ZIP transporters and group F bZIP transcription factors in the Zn-deficiency response of wheat (Triticum aestivum). *Plant J.* 92 291–304. 10.1111/tpj.13655 28771859 PMC5656842

[B20] Gouy M. Guindon S. Gascuel O. (2010). SeaView Version 4: A Multiplatform Graphical User Interface for Sequence Alignment and Phylogenetic Tree Building. *Mol. Biol. Evol.* 27 221–224. 10.1093/molbev/msp259 19854763

[B21] Grotz N. Fox T. Connolly E. Park W. Guerinot M. L. Eide D. (1998). Identification of a family of zinc transporter genes from Arabidopsis that respond to zinc deficiency. *Proc. Natl. Acad. Sci. U. S. A.* 95 7220–7224. 10.1073/pnas.95.12.7220 9618566 PMC22785

[B22] Guerinot M. L. (2000). The ZIP family of metal transporters. *Biochim. Biophys. Acta Biomembr.* 1465 190–198. 10.1016/S0005-2736(00)00138-3 10748254

[B23] Ibrahim E. A. Ramadan W. A. (2015). Effect of zinc foliar spray alone and combined with humic acid or/and chitosan on growth, nutrient elements content and yield of dry bean (Phaseolus vulgaris L.) plants sown at different dates. *Sci. Hortic.* 184 101–105. 10.1016/j.scienta.2014.11.010

[B24] Jefferson R. A. Kavanagh T. A. Bevan M. W. (1987). GUS fusions: beta-glucuronidase as a sensitive and versatile gene fusion marker in higher plants. *EMBO J.* 6 3901–3907. 10.1002/j.1460-2075.1987.tb02730.x 3327686 PMC553867

[B25] Kumar S. Pandey G. (2020). Biofortification of pulses and legumes to enhance nutrition. *Heliyon* 6:e03682. 10.1016/j.heliyon.2020.e03682 32258500 PMC7114740

[B26] Lee S. Lee J. Ricachenevsky F. K. Punshon T. Tappero R. Salt D. E. (2021). Redundant roles of four ZIP family members in zinc homeostasis and seed development in Arabidopsis thaliana. *Plant J.* 108 1162–1173. 10.1111/tpj.15506 34559918 PMC8613002

[B27] Lilay G. H Castro P. H. Campilho A. Assunção A. G. L. (2019). The Arabidopsis bZIP19 and bZIP23 Activity Requires Zinc Deficiency – Insight on Regulation From Complementation Lines. *Front. Plant Sci.* 9:1955. 10.3389/fpls.2018.01955 30723487 PMC6349776

[B28] Lilay G. H Persson D. P. Castro P. H. Liao F. Alexander R. D. Aarts M. G. M. (2021). Arabidopsis bZIP19 and bZIP23 act as zinc sensors to control plant zinc status. *Nat. Plants* 7 137–143. 10.1038/s41477-021-00856-7 33594269

[B29] Lilay G. H. Castro P. H. Guedes J. G. Almeida D. M. Campilho A. Azevedo H. (2020). Rice F-bZIP transcription factors regulate the zinc deficiency response. *J. Exp. Bot.* 71 3664–3677. 10.1093/jxb/eraa115 32133499 PMC7307843

[B30] Lin Y.-F. Liang H.-M. Yang S.-Y. Boch A. Clemens S. Chen C.-C. (2009). Arabidopsis IRT3 is a zinc-regulated and plasma membrane localized zinc/iron transporter. *New Phytol.* 182 392–404. 10.1111/j.1469-8137.2009.02766.x 19210716

[B31] Livak K. J. Schmittgen T. D. (2001). Analysis of Relative Gene Expression Data Using Real-Time Quantitative PCR and the 2–ΔΔCT Method. *Methods* 25 402–408. 10.1006/meth.2001.1262 11846609

[B32] López-Millán A.-F. Ellis D. R. Grusak M. A. (2004). Identification and Characterization of Several New Members of the ZIP Family of Metal Ion Transporters in Medicago Truncatula. *Plant Mol. Biol.* 54 583–596. 10.1023/B:PLAN.0000038271.96019.aa 15316291

[B33] Maret W. Li Y. (2009). Coordination Dynamics of Zinc in Proteins. *Chem. Rev.* 109 4682–4707. 10.1021/cr800556u 19728700

[B34] Mäser P. Thomine S. Schroeder J. I. Ward J. M. Hirschi K. Sze H. (2001). Phylogenetic relationships within cation transporter families of Arabidopsis. *Plant Physiol.* 126 1646–1667. 10.1104/pp.126.4.1646 11500563 PMC117164

[B35] Miller M. A. Pfeiffer W. Schwartz T. (2011). “The CIPRES science gateway,” in *Proceedings of the 2011 TeraGrid Conference on Extreme Digital Discovery - TG ’11*, (New York, NY: ACM Press), 1. 10.1145/2016741.2016785

[B36] Milner M. J. Seamon J. Craft E. Kochian L. V. (2013). Transport properties of members of the ZIP family in plants and their role in Zn and Mn homeostasis. *J. Exp. Bot.* 64 369–381. 10.1093/jxb/ers315 23264639 PMC3528025

[B37] Nazri A. Z. Griffin J. H. C. Peaston K. A. Alexander-Webber D. G. A. Williams L. E. (2017). F-group bZIPs in barley-a role in Zn deficiency. *Plant Cell Environ.* 40 2754–2770. 10.1111/pce.13045 28763829 PMC5656896

[B38] Palmer C. M. Hindt M. N. Schmidt H. Clemens S. Guerinot M. Lou. (2013). MYB10 and MYB72 Are Required for Growth under Iron-Limiting Conditions. *PLoS Genet.* 9:e1003953. 10.1371/journal.pgen.1003953 24278034 PMC3836873

[B39] Robinson G. H. J. Balk J. Domoney C. (2019). Improving pulse crops as a source of protein, starch and micronutrients. *Nutr. Bull.* 44 202–215. 10.1111/nbu.12399 31598097 PMC6772023

[B40] Sinclair S. A. Krämer U. (2012). The zinc homeostasis network of land plants. *Biochim. Biophys. Acta* 1823 1553–1567. 10.1016/j.bbamcr.2012.05.016 22626733

[B41] Vallee B. L. Auld D. S. (1990). Active-site zinc ligands and activated H2O of zinc enzymes. *Proc. Natl. Acad. Sci. U. S. A.* 87 220–224. 10.1073/pnas.87.1.220 2104979 PMC53233

[B42] Vinson C. Acharya A. Taparowsky E. J. (2006). Deciphering B-ZIP transcription factor interactions *in vitro* and *in vivo*. *Biochim. Biophys. Acta* 1759 4–12. 10.1016/j.bbaexp.2005.12.005 16580748

[B43] Wessells K. R. Brown K. H. (2012). Estimating the Global Prevalence of Zinc Deficiency: Results Based on Zinc Availability in National Food Supplies and the Prevalence of Stunting. *PLoS One* 7:e50568. 10.1371/journal.pone.0050568 23209782 PMC3510072

[B44] Yang O. Popova O. V. Süthoff U. Lüking I. Dietz K.-J. Golldack D. (2009). The Arabidopsis basic leucine zipper transcription factor AtbZIP24 regulates complex transcriptional networks involved in abiotic stress resistance. *Gene* 436 45–55. 10.1016/j.gene.2009.02.010 19248824

