# Supplementary material for: Regulation of the Zinc Deficiency Response in the Legume Model Medicago truncatula
Source: Front Plant Sci. 2022 Jun 30;13:916168. doi: 10.3389/fpls.2022.916168 (PMC9279927; doi:10.3389/fpls.2022.916168)
Supplement: Supplementary file 1 [file Data_Sheet_1.pdf]

Supplementary Material

Regulation of the Zinc Deficiency Response in the Legume Model *Medicago truncatula*

Feixue Liao[1], Grmay Hailu Lilay[1], Pedro Humberto Castro[2,3], Herlander Azevedo[2, 3,4], Ana G.L. Assunção[1, 2]

[1] Department of Plant and Environmental Sciences, University of Copenhagen, Frederiksberg, Denmark, [2] CIBIO-InBIO, Research Centre in Biodiversity and Genetic Resources, University of Porto, Vairão, Portugal, [3] BIOPOLIS Biodiversity and Land Planning, Vairão, Portugal, [4] Departamento de Biologia, Faculdade de Ciências, Universidade do Porto, Porto, Portugal

Supplementary Material includes:

Figure S1. Element analysis of *M. truncatula* plants.

Figure S2. Raw data of *M. truncatula* RNA-seq expression atlas.

Figure S3. Transcript level analysis of Arabidopsis transgenic lines.

Figure S4. Analysis of hydroponic-grown Arabidopsis lines.

Table S1. Sequence and ID of F-bZIP homologs enriched in Fabaceae species.

Table S2. Analysis of detected ZDRE motifs in the promoter of annotated *ZIP* genes.

Table S3. List of primers used in this study.

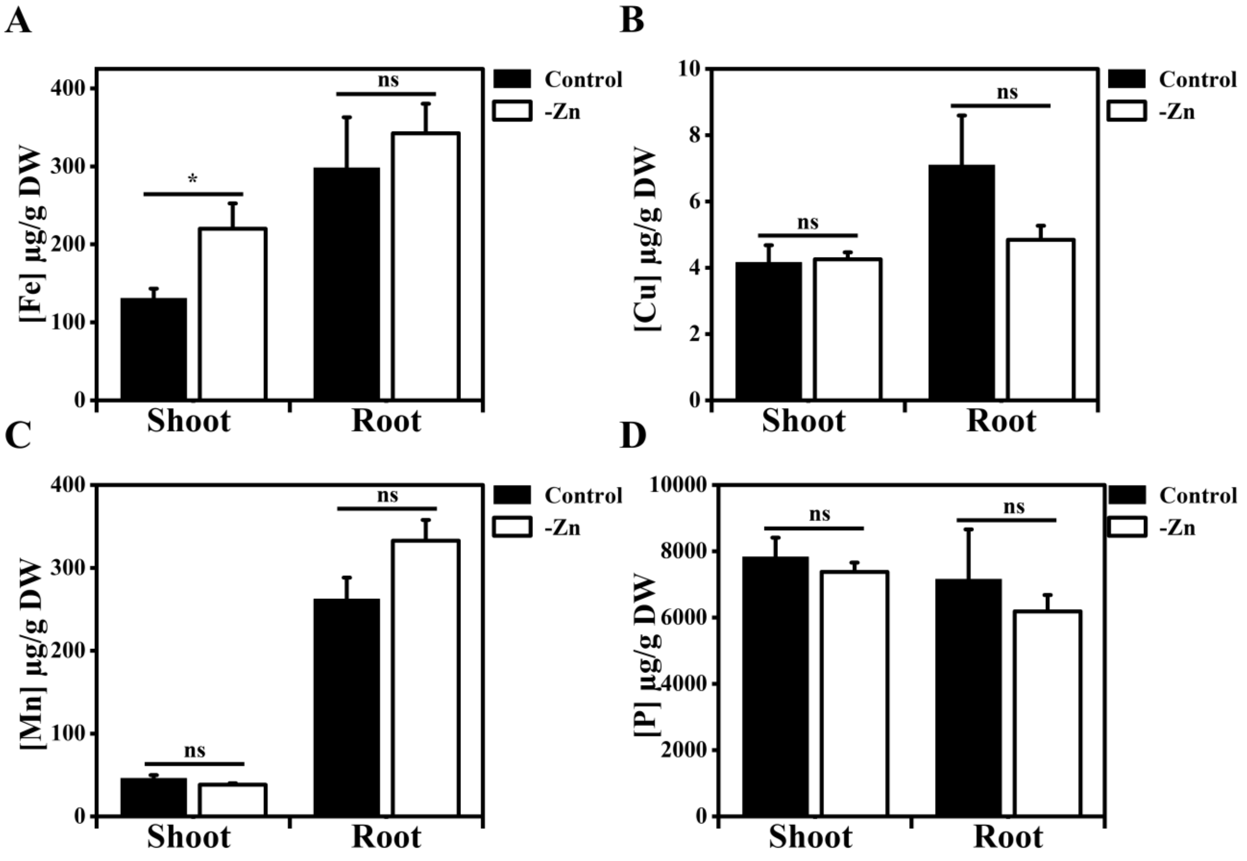

Figure S1. Element analysis of shoots and roots of 6-week-old *M. truncatula* plants grown on sand with nutrient solution at Zn sufficiency (Control) or Zn deficiency (–Zn). (**A-D**) Bars represent (**A**) Fe concentration, (**B**) Cu concentration, (**C**) Mn concentration and (**D**) P concentration. Data represent mean ±SE (n=4). Significant differences between Control and –Zn treatments were determined by Student's t-test (* $p < 0.05$, ns means not significant).

A

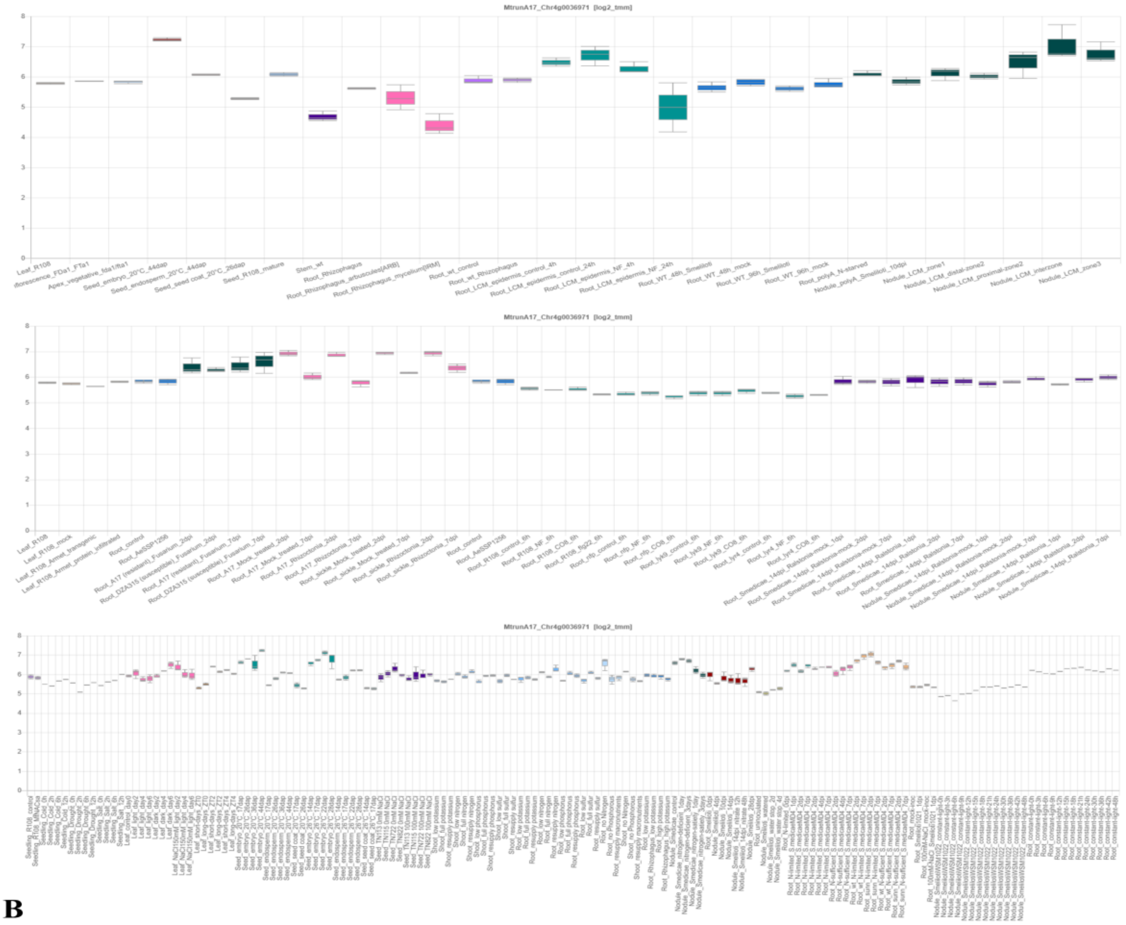
MtrunA17_Chr4g0036971 [log2_tmm]
MtrunA17_Chr4g0036971 [log2_tmm]
MtrunA17_Chr4g0036971 [log2_tmm]

B

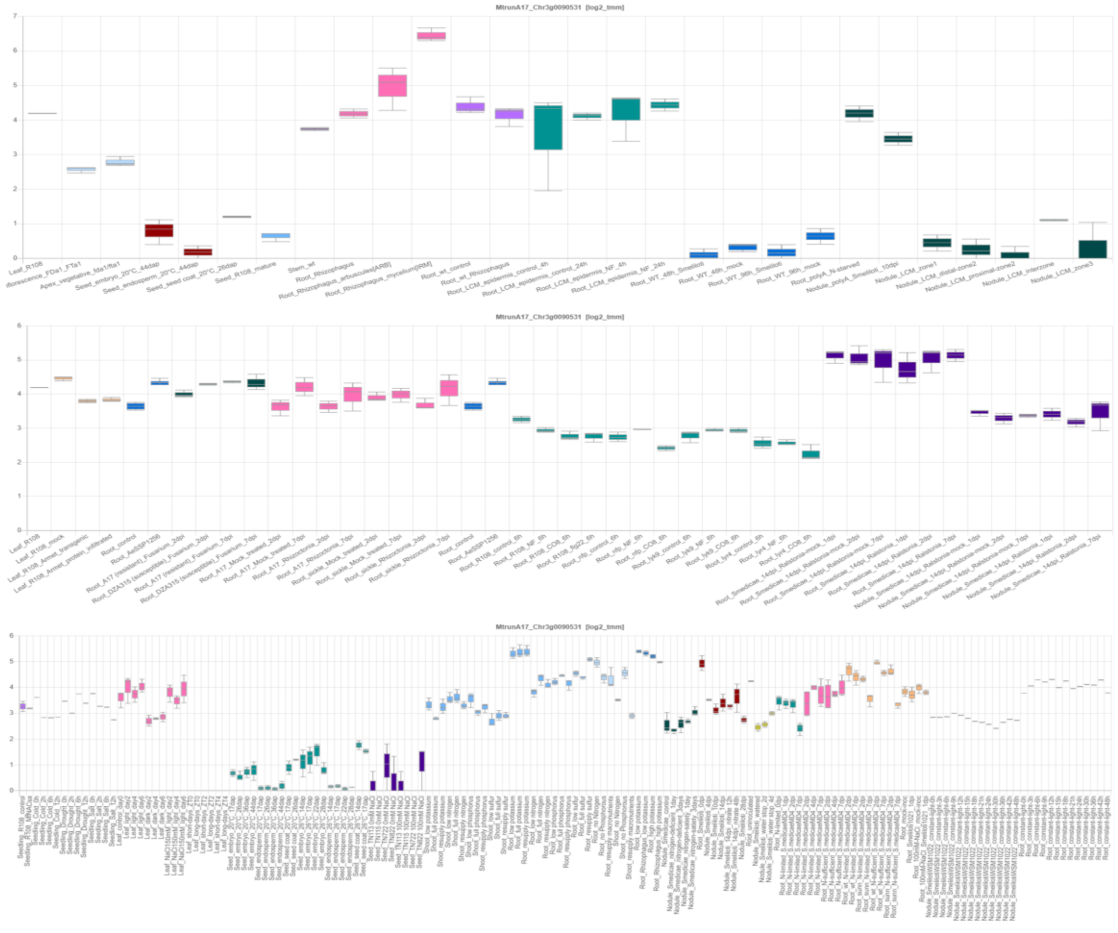
MtrunA17_Chr3g0090531 [log2_tmm]
MtrunA17_Chr3g0090531 [log2_tmm]
MtrunA17_Chr3g0090531 [log2_tmm]

Figure S2. Raw expression data of (**A**) *MtFbZIP1* (MtrunA17_Chr4g0036971) and (**B**) *MtFbZIP2* (MtrunA17_Chr3g0090531) from the datasets: reference/development (upper panel), biotic stress (middle panel) and abiotic factors (lower panel). Graphs were retrieved from the *M. truncatula* expression atlas (https://medicago.toulouse.inrae.fr/MtExpress). Log2_TMM was used to represent normalized relative gene expression values for each gene.

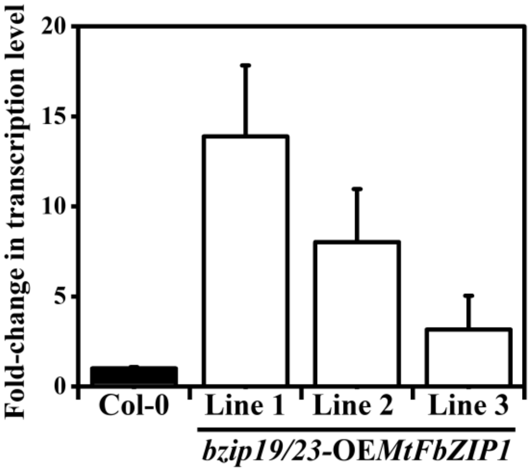

Figure S3. Transcript level profile of *AtbZIP19* (dark bar) and *MtFbZIP1* (open bar) genes in 2-week-old Arabidopsis seedlings of wild-type (Col-0) and *bzip19/23-OEMtFbZIP1* lines, respectively, grown on MS medium. Independently transformed lines of *bzip19/23-OEMtFbZIP1* are designated as Line 1-3. Real-time quantitative RT-PCR was used to determine the expression levels. Bars represent mean fold-change in transcript level ±SE (n=3).

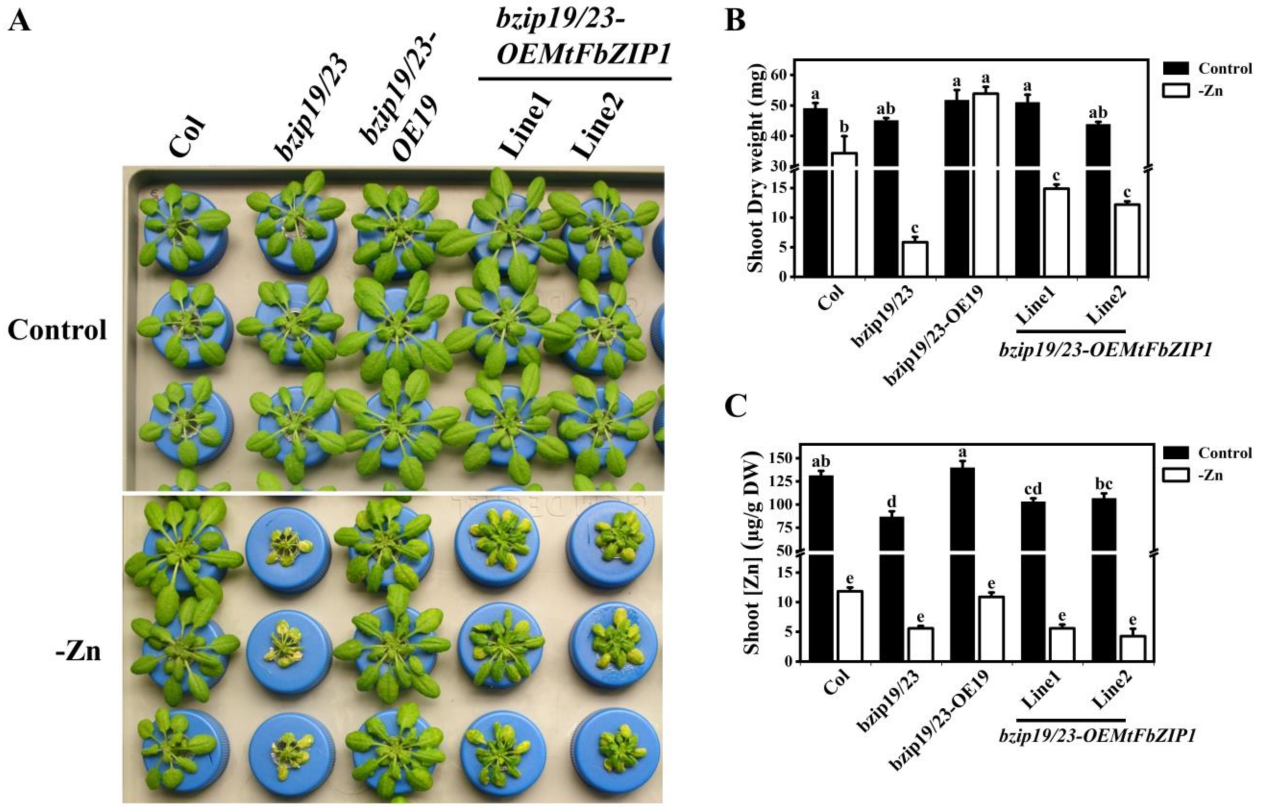

Figure S4. Complementation analysis with *bzip19/23-OEMtFbZIP1* lines, wild-type (Col), *bzip19/23* double mutant and *bzip19/23-OEAtbZIP19* (*bzip19/23-OE19*; Lilay et al., 2019) gown in hydroponics under Zn sufficiency (Control) or deficiency (-Zn). Two independently transformed lines of *bzip19/23-OEMtFbZIP1* line are designated Line 1–2. (**A**) Phenotype of 5-week-old plants. (**B, C**) Shoot dry weight (**B**) and shoot Zn concentration (**C**) in 6-week-old plants. Data represent mean ±SE (n=3-6). Different letters indicate significant differences (p < 0.05) after one-way ANOVA followed by Tukey's post-hoc test.

Table S2. Analysis of detected ZDRE motifs in the gene promoter sequence of annotated *Medicago truncatula*, *Arabidopsis thaliana* and *Amborella trichopoda ZIP* genes and *M. truncatula NAS* genes. [a] Position of the first nucleotide of the ZDRE motif in relation to the start codon. [b] Number of mismatches from the ZDRE consensus sequence (RTGWCGACAY). Mismatches are underlined in the motif sequence. Motifs with no mismatch are in bold. NP stands for None Present and ND stands for No Data.

	PLAZA ID	Gene ID	ZDRE motif	Motif position [a]	Mismatch [b]
MtZIPs	Medtr2g064310	*MtZIP1*	NP		
	Medtr2g097580	*MtZIP2*	NP		
	**Medtr3g081580**	***MtZIP3***	**GTGTCGACAT**	**-282**	**0**
	Medtr3g082050	*MtZIP4*	ATGACAACAT	-2950	1
	**Medtr1g016120**	***MtZIP5***	ATGTCGAAAT	-418	1
			**ATGTCGACAT**	**-407**	**0**
	Medtr4g083570	*MtZIP6*	ATGTCGATAT	-746	1
	Medtr3g058630	*MtZIP7*	NP		
	Medtr3g081640	*MtZIP9*	ATGTTGACAT	-314	1
	Medtr3g081690	*MtZIP10*	ATGACAACAT	-2949	1
	**Medtr3g104400**	***MtZIP11***	**ATGTCGACAC**	**-537**	**0**
			**ATGTCGACAC**	**-452**	**0**
	Medtr5g071990	*MtZIP13*	NP		
	Medtr6g007687	*MtZIP14*	NP		
	Medtr8g105030	*MtZIP16*	ATATCGACAC	-943	1
	Medtr3g081630	*MtZIP17*	NP		
	Medtr4g006710	*MtZIP18*	NP		
MtNASs	Medtr1g084050	*MtNAS1*	GTGTGGACAC	-1103	1
	Medtr2g070310	*MtNAS2*			
	Medtr7g112130	*MtNAS3*			
	**Medtr2g034240**	***MtNAS4***	**ATGTCGACAT**	**-1067**	**0**
AtZIPs	**AT3G12750**	***AtZIP1***	**ATGACGACAT**	**-697**	**0**
	AT5G59520	*AtZIP2*	NP		
	**AT2G32270**	***AtZIP3***	**ATGTCGACAT**	**-1566**	**0**
	**AT1G10970**	***AtZIP4***	**ATGTCGACAT**	**-653**	**0**
			**ATGTCGACAC**	**-525**	**0**
	**AT1G05300**	***AtZIP5***	ATATCGACAT	-1067	1
			**ATGTCGACAT**	**-972**	**0**
	AT2G30080	*AtZIP6*	NP		

	AT2G04032	*AtZIP7*	GTGACGTCAC	-210	1
	**AT4G33020**	***AtZIP9***	**ATGACGACAT**	**-579**	**0**
	**AT1G31260**	***AtZIP10***	**ATGTCGACAC**	**-117**	**0**
	AT1G55910	*AtZIP11*	NP		
	**AT5G62160**	***AtZIP12***	ATGTCGTCAT	-251	1
			**ATGTCGACAT**	**-167**	**0**
	AT4G19690	*AtIRT1*	NP		
	AT4G19680	*AtIRT2*	NP		
	**AT1G60960**	***AtIRT3***	**ATGTCGACAT**	**-264**	**0**
			**ATGTCGACAT**	**-166**	**0**
ATRZIPs	ATR0763G124		ND		
	ATR0752G118		NP		
	ATR0763G166		ATGTCGAGAT	-1483	1
	ATR0681G121		ATGTCGATAT	-691	1
	ATR0752G226		TTGTCGACAT	-404	1
			TTGTCGACAT	-373	1
			ATGACAACAT	-286	1
	**ATR0789G121**		ATGTCGTCAT	-1184	1
			**GTGTCGACAC**	**-306**	**0**
	ATR0747G001		ND		
	**ATR0763G236**		**ATGTCGAGAT**	**-1440**	**0**
	ATR0088G007		NP		
	ATR0763G255		GTGGCGACAT	-1650	1
			ATGTCGCCAC	-508	1
	ATR0752G238		ATGTCGAAAT	-1723	1
	ATR0763G238		ATGACAACAT	-1669	1
			TTGTCGACAT	-1556	1
	ATR0559G300		NP		
	ATR0624G047		NP		

Table S3. Forward (F) and reverse (R) primers used for amplification of *MtFbZIP1 and MtFbZIP2* CDS and for real-time quantitative RT-PCR to analyze the transcript levels of the *M. truncatula* genes: *MtFbZIP1*, *MtFbZIP2*, *MtZIP1*, *MtZIP2*, *MtZIP5*, *MtZIP11*, *MtNAS1-4* and the reference gene *MtUBQ*; and the Arabidopsis genes: *AtbZIP19*, *AtZIP4*, *AtZIP5* and reference the gene *AtACT2*. Restriction enzymes *Not*I and *Asc*I in forward and reverse *MtFbZIP1* primers, respectively, are indicated in bold.

**Primer name**	**Sequence 5'-3'**
MtFbZIP1-F	AT**GCGGCCGC**CATGGAAGACGGTGAACTTGA
MtFbZIP1-R	TA**GGCGCGCC**CCCCGGCTTTGGCTGCAC
MtFbZIP2-F	CACCATGGATGATGGAAATTCTGAG
MtFbZIP2-R	CTCATGTGGTTTGCAATATG
MtUBQ qPCR-F	AAGGCACACAAGTAGGGCAA
MtUBQ qPCR-R	CCTTCAGGATGGTGGAACCC
MtFbZIP1 qPCR-F	ATTGCCTTCGTCCAGATGGG
MtFbZIP1 qPCR-R	ACCACGAAGATCCTTGAGCC
MtFbZIP2 qPCR-F	GAAACTACAAGGGCAGGCAC
MtFbZIP2 qPCR-R	CATGTGGATGCAAACATGGCAG
MtZIP1 qPCR F1	GGCTGCATATCTCAGGCCAA
MtZIP1 qPCR R1	ACTCCCATCCCAATTGCTATCC
MtZIP2 qPCR F1	AGTTGGCGATGGATGAGAGC
MtZIP2 qPCR R1	TTCCAACGGCTATGCCTTCA
MtZIP5 qPCR F1	GGTGTGGATAGTAGCGTCCG
MtZIP5 qPCR R1	TGCAACCACCAAGTGCAAAC
MtZIP11 qPCR F1	TTCTCAGGTGTTAGAGCTTGGG
MtZIP11 qPCR R1	AATGCACCCTCCAAGAGCAA
AtACT2 qPCR-F	CTAAGCTCTCAAGATCAAAGGCTTA
AtACT2 qPCR-R	ACTAAAACGCAAAACGAAAGCGGTT
AtbZIP19 qPCR-F	TTCTCCCGGATGAGAGCGATGA
AtbZIP19 qPCR-R	GCTGATTCACCGCCCTAAGCCT
AtZIP4 qPCR-F	GAGACTCTCTCTCAGAGTCCAT
AtZIP4 qPCR-R	ACCAGCAGCTCCAGCTAGTA
AtZIP5 qPCR-F	CGGGATTGTTGGCGTGGAAT
AtZIP5 qPCR-R	CCAAGACCCTCGAAGCATTG
